# Could Fibroblast Activation Protein (FAP)-Specific Radioligands Be Considered as Pan-Tumor Agents?

**DOI:** 10.1155/2022/3948873

**Published:** 2022-02-22

**Authors:** Hessamoddin Roustaei, Zahra Kiamanesh, Emran Askari, Ramin Sadeghi, Kamran Aryana, Giorgio Treglia

**Affiliations:** ^1^Nuclear Medicine Research Center, Mashhad University of Medical Sciences, Mashhad, Iran; ^2^Ente Ospedaliero Cantonale, Bellinzona, Switzerland; ^3^Faculty of Biomedical Sciences, Universitá della Svizzera italiana, Lugano, Switzerland; ^4^Faculty of Biology and Medicine, University of Lausanne, Lausanne, Switzerland

## Abstract

**Background:**

Cancer-associated fibroblasts (CAFs) can strongly modulate the response to therapy of malignant tumor cells, facilitating their continuous proliferation and invading behaviors. In this context, several efforts were made in identifying the fibroblast activation protein (FAP) as a CAF recognizer and in designing FAP-specific PET radiotracers (as ^68^Ga-FAPI) along with FAP-specific therapeutic radioligands. Herein, we review different clinical studies using the various FAP-specific radioligands as novel theranostic agents in a wide range of oncologic and nononcologic indications.

**Methods:**

A comprehensive systematic search was conducted on the PubMed and Scopus databases to find relevant published articles concerning the FAP-specific PET imaging as well as the FAP-specific radionuclide therapy in patients with oncologic and nononcologic indications. The enrolled studies were dichotomized into oncologic and nononcologic categories, and the required data were extracted by precisely reviewing the whole text of each eligible study. A meta-analysis was also performed comparing the detection rates of ^68^Ga-FAPI vs. ^18^F-FDG PET/CT using odds ratio (OR) and risk difference as outcome measures.

**Results:**

Of the initial 364 relevant papers, 49 eligible articles (1479 patients) and 55 case reports were enrolled in our systematic review. These studies observed high radiolabeled FAPI avidity as early as 10 minutes after administration in primary sites of various malignant tumors. Based on the meta-analysis which was done on the reported detection rates of the ^68^Ga-FAPI and ^18^F-FDG PET/CT scans, the highest OR belonged to the primary lesion detection rate of gastrointestinal tumors (OR = 32.079, 95% CI: 4.001–257.212; *p* = 0.001) with low heterogeneity (I^2^ = 0%). The corresponding value of the nodal metastases belonged to hepatobiliary tumors (OR = 11.609, 95% CI: 1.888–71.365; *p* = 0.008) with low heterogeneity (I^2^ = 0%). For distant metastases, the highest estimated OR belonged to nasopharyngeal carcinomas (OR = 77.451, 95% CI: 7.323–819.201; *p* < 0.001) with low heterogeneity (I^2^ = 0%).

**Conclusions:**

The outperformance of ^68^Ga-FAPI PET/CT over ^18^F-FDG PET/CT in identifying certain primary tumors as well as in detecting their metastatic lesions may open indications for evaluation of cases with inconclusive ^18^F-FDG PET/CT findings. What needs to be emphasized is that the false-positive results might be problematic and must be taken into account in ^68^Ga-FAPI PET/CT interpretation. More clarification on the role of FAPI radioligands in oncologic imaging, radionuclide therapy, and radiotherapy treatment planning is therefore required.

## 1. Introduction

Despite the occurrence of major developments in the diagnosis and treatment of malignant neoplasms, cancer remains the second leading cause of death, accounting for nearly 10 million deaths in 2020 [1]. This matter of fact has intensified the efforts of investigators to solve this clinical problem. In this regard, the cancer-promoting role of the tumor microenvironment (TME) is one of the issues that has recently gained scientists' interest. The tumor stroma constitutes a major part of the tumoral lesion and has common elements between various types of cancers. Apart from tumor cells, TME recruits various nonmalignant cells (comprising immune cells, endothelial cells, epithelial cells, fibroblasts, and adipocytes), which coordinate with each other via a complex and dynamic network of different cytokines and chemokines [2, 3]. Observations indicate that the genetically stable cells residing in the TME can strongly modulate response to therapy of the malignant mutant cells and facilitate their continuous proliferation and invading behaviors [2–7].

Cancer-associated fibroblasts (CAFs) are known as important drivers of stromal interactions. The CAF's cancer-promoting roles have been attributed to their diverse secretome. These cells can produce a cancer-specific extracellular matrix (ECM) as well as various soluble factors such as growth factors, cytokines, and enzymes. This secretome makes the CAFs capable of remodeling the ECM, local invasion, distant migration, uninterrupted proliferation, angiogenesis, tumor stiffness, and modulating the immune response and tumor response to therapy [4–7].

Targeting CAFs is an attractive purpose for functional imaging. Besides, altering their numbers or functionality can be an exciting goal to improve the therapeutic perspective. In this context, identifying the fibroblast activation protein (FAP) as a CAF recognizer and designing FAP-specific PET radiotracers along with FAP-specific therapeutic radioligands are some of the consequences of the efforts made.

In the current study, we reviewed the different clinical studies using the various FAP and FAP inhibitor (FAPI)-specific radioligands as novel theranostic agents in a wide spectrum of oncologic and nononcologic indications.

## 2. Methods

### 2.1. Literature Search

A comprehensive systematic search was conducted on the PubMed and Scopus databases to find relevant published articles concerning the FAP-specific PET imaging in patients with oncologic and nononcologic indications. The search strategy was (“FAP” OR “FAPI” OR “fibroblast activation protein”) AND (“PET” OR “positron emission tomography” OR “SPECT”). The search was not restricted to a specific date or language and was updated until May 2021.

### 2.2. Eligibility Criteria

Investigations related to the FAP-specific PET imaging in patients with oncologic and nononcologic indications were considered for inclusion, and studies in the preclinical phase, review articles, or letters to editors were excluded. In the first step, title and abstract screening of the retrieved articles was done. In the next step, the full-text version assessment of the remaining papers was done to verify their eligibility for inclusion. The reference lists of the pertinent articles were also retrieved to identify any other relevant papers. Articles cited in the included studies were also checked using Google Scholar.

All studies which compared ^68^Ga-FAPI with ^18^F-FDG, regarding detection rates of the primary lesions and nodal and distant metastases were included in the meta-analysis (if they provided enough quantitative data).

### 2.3. Data Extraction

The enrolled studies were dichotomized in oncologic and nononcologic clusters, and the required data were extracted by precisely reviewing the whole text of each eligible study. Eventually, the gathered data were categorized into three main parts: (i) Basic study characteristics consist of the first author's name and year of publication. (ii) Demographic characteristics consist of the type of cancerous or noncancerous disease and the number of participants. (iii) Methodological aspects consist of used radiotracer ligands, radioisotopes, and imaging method.

### 2.4. Statistical Analysis

Meta-analysis was carried out using comprehensive meta-analysis software (CMA version 2) in a random-effects model. The outcome variables were the odds ratio and risk difference between the detection rate of ^68^Ga-FAPI and ^18^F-FDG PET/CT scans [8]. Heterogeneity was evaluated by Cochrane Q value (*p* < 0.05 was considered statistically significant) as well as the I^2^ (inconsistency index).

## 3. Results

### 3.1. Systematic Review

**Figure 1:**
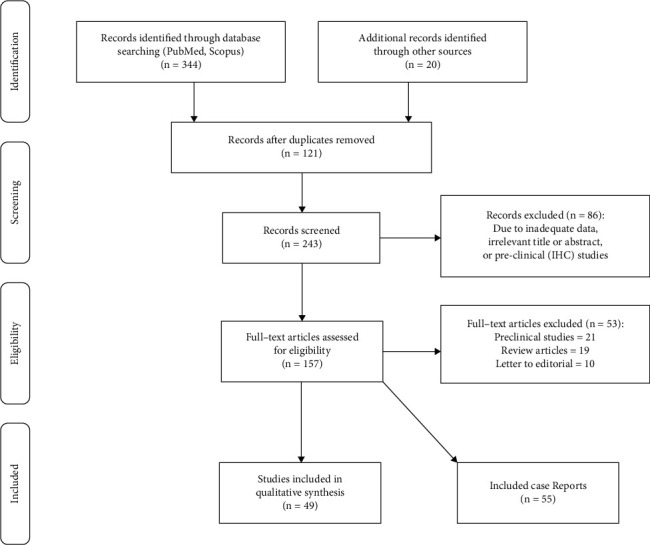
PRISMA flow chart of the adopted search strategy.

In the present systematic review, a total of 344 relevant records were retrieved from PubMed and Scopus databases. Besides, twenty additional records were identified through reference list evaluation and forward citation analysis using Google Scholar. The adopted strategy is illustrated in Figure 1 as a PRISMA flow chart [9]. According to the title and abstract screening, 86 irrelevant articles were excluded. In the next stage, the full-text version of the remaining studies was assessed thoroughly and 53 studies were also excluded. Ultimately, 49 eligible articles were included for data extraction. The enrolled studies were dichotomized into oncologic (41 papers) and nononcologic (8 papers) categories. Basic study characteristics of eligible original articles are summarized in Supplementary Table 1, in two parts: oncologic and nononcologic applications. Focusing on the presently enrolled studies reveals a variety of ligands (FAPI-02, FAPI-04, FAPI-05, FAPI-34, FAPI-46, FAPI-74, and FAPI-2286), chelating agents (DOTA, DATA, and NOTA), and radioisotopes (^68^Ga, ^18^F, ^99m^Tc, and ^177^Lu). Advantages and disadvantages of various clinically used FAPI radioligands are tabulated in Supplementary Table 2. The majority of the published papers were categorized as case reports. We have summarized 55 case reports in this review. The case report findings are reported in Supplementary Table 3.

### 3.2. Meta-Analysis

**Figure 2:**
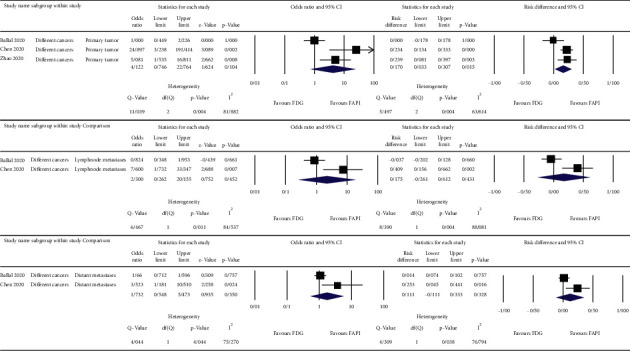
OR, risk differences and related heterogeneity indices of detection rates of radiolabeled FAPI guided and ^18^F-FDG directed PET/CT scans for different cancers in primary tumor as well as lymph node and distant metastases.

**Figure 3:**
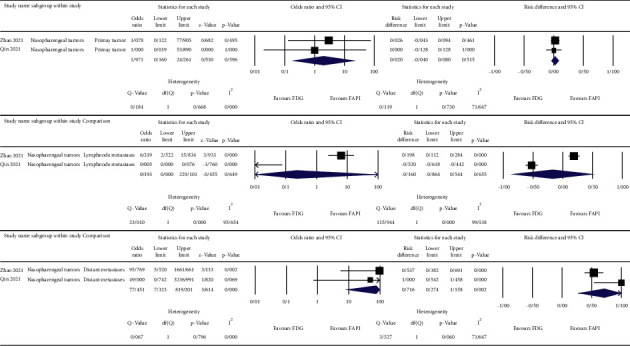
OR, risk differences and related heterogeneity indices of detection rates of radiolabeled FAPI guided and ^18^F-FDG directed PET/CT scans for nasopharyngeal carcinomas in primary tumor as well as lymph node and distant metastases.

**Figure 4:**
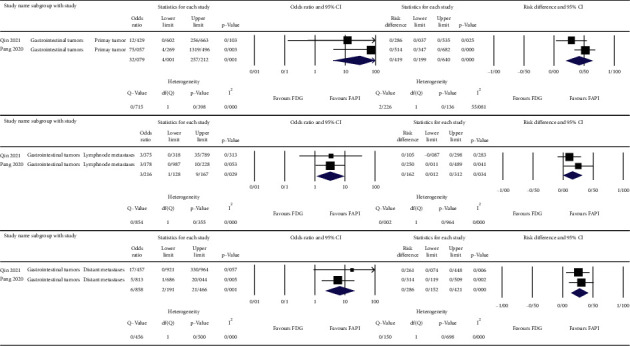
OR, risk differences and related heterogeneity indices of detection rates of radiolabeled FAPI guided and ^18^F-FDG directed PET/CT scans for gastrointestinal carcinomas in primary tumor as well as lymph node and distant metastases.

**Figure 5:**
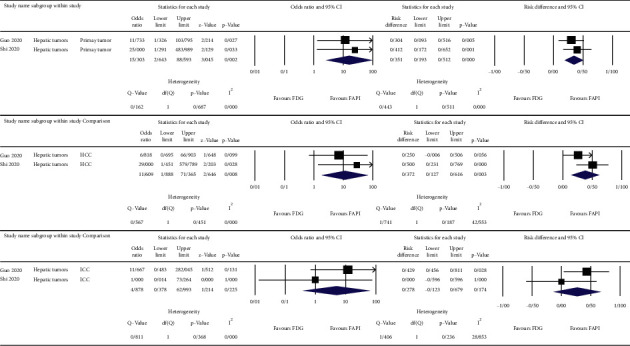
OR, risk differences and related heterogeneity indices of detection rates of radiolabeled FAPI guided and ^18^F-FDG directed PET/CT scans for hepatic tumors.

The meta-analysis was done on the reported detection rates of the ^68^Ga-FAPI and ^18^F-FDG PET/CT scans for different cancer types [10–12], nasopharyngeal carcinomas [13, 14], gastrointestinal tumors [15, 16], and hepatobiliary tumors [17, 18]. For the primary lesions, we used patient-based detection rates, and for the nodal and distant metastases, we used lesion-based detection rates of either ^68^Ga-FAPI or ^18^F-FDG PET/CT scans. The results are depicted as forest plots in Figures 2–5.

The highest estimated OR between the primary tumor detection rates of ^68^Ga-FAPI and ^18^F-FDG PET/CT scans belonged to gastrointestinal tumors (OR = 32.079, 95% CI: 4.001–257.212; *p* = 0.001) with low heterogeneity (I^2^ = 0%) (Figure 4). The corresponding value of the nodal metastases belonged to hepatobiliary tumors (OR = 11.609, 95% CI: 1.888–71.365; *p* = 0.008) with low heterogeneity (I^2^ = 0%) (Figure 5). For distant metastases, the highest estimated OR belonged to nasopharyngeal carcinomas (OR = 77.451, 95% CI: 7.323–819.201; *p* < 0.001) with low heterogeneity (I^2^ = 0%) (Figure 3). On the other hand, the calculations showed high heterogeneity for ORs of different cancer types in primary tumor detection (I^2^ = 81.882), nodal and distant metastases (I^2^ = 84.537 and I^2^ = 75.270, respectively) (Figure 2), and the nodal metastases of nasopharyngeal carcinomas (I^2^ = 95.654) (Figure 3).

## 4. Discussion

### 4.1. Radioligands

Among the introduced FAP-specific radioligands, it seems that the ^68^Ga-FAPI-46 could be an optimal agent for diagnostic imaging due to its rapid and high uptake in malignant lesions as well as low background retention. On the contrary, ^177^Lu-FAPI-2286 has superiority in therapeutic applications because of its long tumor retention until even 10 days.

### 4.2. Oncologic Applications

Targeting fibroblast activation protein is a new diagnostic approach to visualize the stroma of malignant tumors. It seems that radiolabeled FAPI is a promising theranostic agent for oncologic purposes, as it may help to identify new lesions or clarify inconclusive findings obtained by other imaging modalities and may provide a new therapeutic modality. In this context, some malignant tumors exhibit stronger enhancement that is illustrated by patients harboring head and neck cancer, nasopharyngeal carcinoma, non-small-cell lung cancer, hepatocellular carcinoma, cholangiocarcinoma, pancreatic cancer, esophageal cancer, gastric cancer, duodenal cancer, colorectal cancer, anal cancer, breast cancer, cervical cancer, ovarian cancer, some types of lymphoma, and sarcoma [10, 11, 15, 18–30]. Apart from high radiotracer uptake in a tumor lesion, the unique feature of the FAPI radiotracers is the very low background uptake [22–24, 31]. Indeed, the favorable contrast of a FAPI-specific PET/CT scan is attributed to low background uptake that results in a superior target-to-nontarget ratio of even more than 6 [17–19, 21, 25, 27, 32]. The rapid radiotracer uptake and high target-to-background ratio even at 10 min after injection and consequently the possibility of early-time-point ^68^Ga-FAPI imaging can simplify the clinical workflow, reduce the radiation burden of the patients, and cause patient comfort due to shorter waiting and scan time [11, 20, 33, 34]. Another considerable potential advantage of these agents is independence to blood sugar level and no need for dietary preparation [20]. It should be noted that good patient toleration without any symptoms has been frequently reported for FAPI radiotracers [10, 11, 15, 19–23, 33, 35–38].

The outperformance of ^68^Ga-FAPI over ^18^F-FDG PET/CT in identifying primary tumors as well as in detection of metastatic lesions in either newly diagnosed or previously treated tumors was reported by several studies [10, 12, 14–16, 18, 19, 21, 29, 39, 40]. It should be mentioned that normal physiologic glucose metabolism, small tumor size (<1 cm), and partial volume effect could influence the ^18^F-FDG PET/CT performance in visualizing malignant lesions [10]. Notably, low background activity and capacity of visualizing small malignant lesions (<1 cm) could improve the ^68^Ga-FAPI PET/CT performance [10, 17]. The stroma-specific PET imaging may be more sensitive than glycolysis-specific PET imaging in the identification of small lesions with adequate FAP expression. Hence, these potential benefits may open indications for ^68^Ga-FAPI in evaluating cases with inconclusive ^18^F-FDG PET/CT findings and play a complementary role to this traditional oncologic agent in pinpointing the early-stage cancers and the primary site in CUPs [19, 21]. On the contrary, some investigations did not find superiority in the detection rate of ^68^Ga-FAPI PET/CT scan over ^18^F-FDG PET/CT scan for either primary lesion or metastases [11, 13, 20, 32, 41]. It is worth noting that the FAPI-specific PET/CT imaging in thyroid cancers with a flip-flop phenomenon on ^18^F-FDG PET/CT, metastatic castration-resistant prostate cancers with non-PSMA-avid metastases in ^68^Ga-PSMA PET/CT, and unknown primary tumors may be interesting subjects for further investigations.

A newly published meta-analysis reported patient-based pooled sensitivity and specificity of the ^68^Ga-FAPI PET/CT imaging as follows: 99% (95% CI: 0.97–1.00, I^2^ = 0%; *p* = 0.75) and 87% (95% CI: 0.62–1.00, I^2^ = 0%; *p* = 0.51), respectively [42]. The calculated pooled sensitivity for primary tumor detection was 1.00 (95% CI: 0.98–1.00; I^2^ = 0%; *p* = 0.83) [42]. The corresponding value for identifying distant metastases using ^68^Ga-FAPI was 0.93 (95% CI: 0.88–0.97; I^2^ = 0%; *p* = 0.41) [42]. However, their calculated lesion-based pooled sensitivity and specificity for nodal metastases affected by high heterogeneity (I^2^ = 88.56 and I^2^ = 97.20, respectively) were not reliable [42]. Similarly, in our meta-analysis, the estimated ORs of different cancers for primary lesions (I^2^ = 81.882) as well as nodal and distant metastases (I^2^ = 84.537 and I^2^ = 75.270, respectively) were biased by high heterogeneity [42]. Ultimately, they believed ^68^Ga-FAPI-guided PET could be promising, especially in malignancies unsuitable for ^18^F-FDG-directed imaging [42]. This newly published systematic review has several methodological flaws including the following: (1) only a small number of studies were included, (2) the included studies all reported mixed population of different cancers, and different cancers with different clinical behaviors were all pooled together, and (3) there is no discussion on the potential pitfalls of FAPI-directed PET.

### 4.3. Nononcologic Applications

It is quoted that “cancers are wounds never heal” because the stroma of cancer and wound share many similarities, for instance, fibroblast activation and intensive remodeling processes [5]. Activated fibroblasts have an important contribution in an activated stroma; however, it remains to be understood how these activated fibroblasts react to either tumors or wounds and evolve into CAFs or myofibroblasts. Therefore, in addition to malignant tumors, CAFs accompanied with their FAP indicators may be present in nonmalignant situations that induce fibroblast activation. Thus, besides the oncologic indications of the FAPI-guided imaging, some investigators evaluated radiolabeled FAPI avidity in several nononcologic diseases such as acute or chronic heart diseases, IgG4-related diseases, and diseases with predominant fibrotic activity in different organs [38, 43–48]. It should be mentioned that the ^68^Ga-FAPI uptake in nononcologic indications did not necessarily correlate with the ^18^F-FDG signals, which suggested fibrosis and inflammation are not essentially interconnected [38, 46, 48].

In this context, it is demonstrated that focal myocardial ^68^Ga-FAPI avidity could be correlated with older age, lower left ventricular ejection fraction, a higher percentage of significant coronary artery diseases, hypertension, and medication with aspirin or statins [45, 47]. On the contrary, participants without localized uptake showed neither history of coronary artery diseases nor myocardial infarction [45]. Moreover, this imaging modality could allow the identification of local myocardial remodeling due to immune checkpoint-associated myocarditis [44]. In IgG4-related diseases, ^68^Ga-FAPI-04 avidity is not correlated to ^18^F-FDG, suggesting that mesenchymal cell activation is not associated with the hypermetabolic activity of infiltrating immune cells [38, 46]. Lesions could be “silent” on ^18^F-FDG despite “bright” in ^68^Ga-FAPI-04 PET/CT imaging [46]. Therefore, discrimination of inflammatory activities from profibrotic activities is feasible in IgG4-related disease using these two PET tracers [46]. Fibrotic activity in the format of renal fibrosis depicted increased ^68^Ga-FAPI-04 uptake, as well [43]. Furthermore, ^68^Ga-FAPI-04 uptake of fibrotic activity in the form of retroperitoneal fibrosis, cirrhotic livers, fibrous dysplasia, myelofibrosis, elastofibroma dorsi, and solitary fibrous tumor was reported [15, 49–52]. Indeed, FAP-specific PET/CT could be used to detect the fibrotic activity noninvasively and potentially earlier than anatomical imaging techniques in different organs. By the way, the recently published systematic review on nonmalignant indications of FAP-specific PET/CT scan stated further investigations are warranted to clarifying the role of FAP-specific imaging in nononcologic applications especially in cardiology and immunology/rheumatology imaging [53].

### 4.4. Potential Pitfalls

Since the pitfalls and incidental uptakes in FAPI-guided PET/CT have revealed high diversity, it is difficult to categorize them into certain groups. Anyway, the mentioned pitfalls can be classified as follows: (i) activated fibrotic reactions, including fibrotic phase of IgG4-related diseases, MI, posttherapeutic scars, etc. [15, 49–52]; (ii) acute or chronic inflammatory processes including pancreatitis, bone fractures, tuberculosis, cirrhosis, etc. [15, 17, 18, 21, 25, 31, 54–63]; (iii) benign tumors including hemangioma, angiomyolipoma, thyroid adenoma, elastofibroma dorsi, etc. [17, 51, 64, 65]. Pitfalls related to the breast and uterus normal tissues were attributed to hormonal changes or physiologic uptake, postpartum changes, mastitis, and localized benign lymphoid tissue [30, 31, 66–69]. The potential pitfalls might be problematic in reading ^68^Ga-FAPI PET/CT images and must be taken into consideration during the scan interpretation [21]. In these conditions, careful attention to morphological characteristics in CT scans or MRI and clinical data may help differentiate between false positives and true malignancies [17].

### 4.5. Therapeutic Applications

In line with published results, high FAPI avidity, low background activity, encouraging contrast, prolonged tumor retention, and chelators capable of linking with a therapeutic radionuclide could be considered as favorable properties for potential therapeutic applications [20, 33, 70–73]. However, further investigations and prospective clinical studies are required to optimize therapeutic efficacy. On the other hand, FAP-specific PET imaging represented promising results for GTV contouring in head and neck cancer, nasopharyngeal carcinoma, adenoid cystic carcinoma, glioblastoma, and esophageal cancer [13, 22, 24, 35, 40, 74]. Regarding pancreatic cancer and lung cancer, there was a controversy and no preference was found compared to conventional methods [37, 75]. For more clarification of the competence of FAP-specific imaging in radiotherapy planning, further research studies are warranted, as was stated by another published systematic review in this field [76].

## 5. Conclusion

The FAP-specific radiotracers are not a perfect pan-tumor agent, but some of the properties that make them unique are their high avidity to a wide range of malignant tumors, low background activity, and favorable image contrast.

Apart from FAPI imaging in the IgG4-related disease with encouraging results, other nononcologic purposes are still premature to draw any conclusion. The outperformance of ^68^Ga-FAPI PET/CT in the detection of primary tumors and nodal or distant metastatic lesions (especially in nasopharyngeal, hepatic, and gastrointestinal malignancies) opens indications for ^68^Ga-FAPI to have a complementary role in ^18^F-FDG PET/CT imaging.

What needs to be emphasized is that the potential pitfalls might be problematic and must be taken into consideration in ^68^Ga-FAPI PET/CT interpretation. Eventually, further investigations on diagnostic FAPI radiotracers, potential pitfalls, FAPI-targeted radionuclide therapy, and radiotherapy treatment planning are required.

## Data Availability

Data are available from bibliographic databases.

## Supplementary Materials

Supplementary Materials Supplementary Table 1: summary of clinical studies on FAP-tracers. Supplementary Table 2: characteristics of FAP-tracers. Supplementary Table 3: summary of case reports on FAP-tracers.

## References

[1] Ferlay J., Ervik M., Lam F. (2021). *Global Cancer Observatory: Cancer Today*.

[2] Balkwill F. R., Capasso M., Hagemann T. (2012). The tumor microenvironment at a glance. *Journal of Cell Science*.

[3] Koustoulidou S., Hoorens M. W. H., Dalm S. U. (2021). Cancer-associated fibroblasts as players in cancer development and progression and their role in targeted radionuclide imaging and therapy. *Cancers*.

[4] Sahai E., Astsaturov I., Cukierman E. (2020). A framework for advancing our understanding of cancer-associated fibroblasts. *Nature Reviews Cancer*.

[5] Liu T., Zhou L., Li D., Andl T., Zhang Y. (2019). Cancer-associated fibroblasts build and secure the tumor microenvironment. *Frontiers in Cell and Developmental Biology*.

[6] Franco O. E., Shaw A. K., Strand D. W., Hayward S. W. (2010). Cancer associated fibroblasts in cancer pathogenesis. *Seminars in Cell & Developmental Biology*.

[7] Biffi G., Tuveson D. A. (2021). Diversity and biology of cancer-associated fibroblasts. *Physiological Reviews*.

[8] Zamora J., Abraira V., Muriel A., Khan K., Coomarasamy A. (2006). Meta-DiSc: a software for meta-analysis of test accuracy data. *BMC Medical Research Methodology*.

[9] Moher D., Liberati A., Tetzlaff J., Altman D. G. (2010). Preferred reporting items for systematic reviews and meta-analyses: the PRISMA statement. *International Journal of Surgery*.

[10] Chen H., Pang Y., Wu J. (2020). Comparison of [68Ga]Ga-DOTA-FAPI-04 and [18F] FDG PET/CT for the diagnosis of primary and metastatic lesions in patients with various types of cancer. *European Journal of Nuclear Medicine and Molecular Imaging*.

[11] Ballal S., Yadav M. P., Moon E. S. (2020). Biodistribution, pharmacokinetics, dosimetry of [68Ga]Ga-DOTA.SA.FAPi, and the head-to-head comparison with [18F]F-FDG PET/CT in patients with various cancers. *European Journal of Nuclear Medicine and Molecular Imaging*.

[12] Zhao L., Pang Y., Luo Z. (2021). Role of [68Ga]Ga-DOTA-FAPI-04 PET/CT in the evaluation of peritoneal carcinomatosis and comparison with [18F]-FDG PET/CT. *European Journal of Nuclear Medicine and Molecular Imaging*.

[13] Qin C., Liu F., Huang J. (2021). A head-to-head comparison of 68Ga-DOTA-FAPI-04 and 18F-FDG PET/MR in patients with nasopharyngeal carcinoma: a prospective study. *European Journal of Nuclear Medicine and Molecular Imaging*.

[14] Zhao L., Pang Y., Zheng H. (2021). Clinical utility of [68Ga]Ga-labeled fibroblast activation protein inhibitor (FAPI) positron emission tomography/computed tomography for primary staging and recurrence detection in nasopharyngeal carcinoma. *European Journal of Nuclear Medicine and Molecular Imaging*.

[15] Pang Y., Zhao L., Luo Z. (2020). Comparison of 68Ga-FAPI and 18F-fdg uptake in gastric, duodenal, and colorectal cancers. *Radiology*.

[16] Qin C., Shao F., Gai Y. (2021). 68Ga-DOTA-FAPI-04 PET/MR in the evaluation of gastric carcinomas: comparison with 18F-FDG PET/CT. *Journal of Nuclear Medicine*.

[17] Guo W., Pang Y., Yao L. (2020). Imaging fibroblast activation protein in liver cancer: a single-center post hoc retrospective analysis to compare [68Ga]Ga-FAPI-04 PET/CT versus MRI and [18F]-FDG PET/CT. *European Journal of Nuclear Medicine and Molecular Imaging*.

[18] Shi X., Xing H., Yang X. (2020). Comparison of PET imaging of activated fibroblasts and 18F-FDG for diagnosis of primary hepatic tumours: a prospective pilot study. *European Journal of Nuclear Medicine and Molecular Imaging*.

[19] Kratochwil C., Flechsig P., Lindner T. (2019). 68Ga-FAPI PET/CT: tracer uptake in 28 different kinds of cancer. *Journal of Nuclear Medicine*.

[20] Giesel F. L., Kratochwil C., Lindner T. (2019). 68Ga-FAPI PET/CT: biodistribution and preliminary dosimetry estimate of 2 DOTA-containing FAP-targeting agents in patients with various cancers. *Journal of Nuclear Medicine*.

[21] Chen H., Zhao L., Ruan D. (2020). Usefulness of [68Ga]Ga-DOTA-FAPI-04 PET/CT in patients presenting with inconclusive [18F]FDG PET/CT findings. *European Journal of Nuclear Medicine and Molecular Imaging*.

[22] Syed M., Flechsig P., Liermann J. (2020). Fibroblast activation protein inhibitor (FAPI) PET for diagnostics and advanced targeted radiotherapy in head and neck cancers. *European Journal of Nuclear Medicine and Molecular Imaging*.

[23] Koerber S. A., Staudinger F., Kratochwil C. (2020). The role of 68Ga-FAPI PET/CT for patients with malignancies of the lower gastrointestinal tract: first clinical experience. *Journal of Nuclear Medicine*.

[24] Ristau J., Giesel F. L., Haefner M. F. (2020). Impact of primary staging with fibroblast activation protein specific enzyme inhibitor (FAPI)-PET/CT on radio-oncologic treatment planning of patients with esophageal cancer. *Molecular Imaging and Biology*.

[25] Shi X., Xing H., Yang X. (2020). Fibroblast imaging of hepatic carcinoma with 68Ga-FAPI-04 PET/CT: a pilot study in patients with suspected hepatic nodules. *European Journal of Nuclear Medicine and Molecular Imaging*.

[26] Jin X. (2021). Detecting fibroblast activation proteins in lymphoma using (68)Ga-FAPI PET/CT. *Journal of Nuclear Medicine*.

[27] Koerber S. A., Finck R., Dendl K. (2021). Novel FAP ligands enable improved imaging contrast in sarcoma patients due to FAPI-PET/CT. *European Journal of Nuclear Medicine and Molecular Imaging*.

[28] Linz C., Brands R. C., Kertels O. (2021). Targeting fibroblast activation protein in newly diagnosed squamous cell carcinoma of the oral cavity - initial experience and comparison to [18F]FDG PET/CT and MRI. *European Journal of Nuclear Medicine and Molecular Imaging*.

[29] Kömek H., Can C., Güzel Y. (2021). 68Ga-FAPI-04 PET/CT, a new step in breast cancer imaging: a comparative pilot study with the 18F-FDG PET/CT. *Annals of Nuclear Medicine*.

[30] Dendl K., Koerber S. A., Finck R. (2021). 68Ga-FAPI-PET/CT in patients with various gynecological malignancies. *European Journal of Nuclear Medicine and Molecular Imaging*.

[31] Röhrich M. (2020). Impact of 68Ga-FAPI-PET/CT imaging on the therapeutic management of primary and recurrent pancreatic ductal adenocarcinomas. *Journal of Nuclear Medicine*.

[32] Serfling S., Zhi Y., Schirbel A. (2021). Improved cancer detection in Waldeyer’s tonsillar ring by 68Ga-FAPI PET/CT imaging. *European Journal of Nuclear Medicine and Molecular Imaging*.

[33] Meyer C., Dahlbom M., Lindner T. (2020). Radiation dosimetry and biodistribution of 68Ga-FAPI-46 PET imaging in cancer patients. *Journal of Nuclear Medicine*.

[34] Ferdinandus J., Kessler L., Hirmas N. (2021). Equivalent tumor detection for early and late FAPI-46 PET acquisition. *European Journal of Nuclear Medicine and Molecular Imaging*.

[35] Windisch P., Röhrich M., Regnery S. (2020). Fibroblast Activation Protein (FAP) specific PET for advanced target volume delineation in glioblastoma. *Radiotherapy & Oncology*.

[36] Wang S., Zhou X., Xu X. (2021). Dynamic PET/CT imaging of 68Ga-FAPI-04 in Chinese subjects. *Frontiers in Oncology*.

[37] Giesel F. (2020). FAPI-74 PET/CT using either 18F-AlF or cold-kit 68Ga-labeling: biodistribution, radiation dosimetry and tumor delineation in lung cancer patients. *Journal of Nuclear Medicine*.

[38] Luo Y., Pan Q., Yang H., Peng L., Zhang W., Li F. (2020). Fibroblast activation protein-targeted PET/CT with 68Ga-FAPI for imaging IgG4-related disease: comparison to 18F-fdg PET/CT. *Journal of Nuclear Medicine*.

[39] Bal C. (2020). 68Ga-DATA-FAPi-05: biodistribution and comparison with 18F-fdg PET/CT in various cancers. *Soc Nuclear Med*.

[40] Zhao L., Chen S., Chen S. (2021). 68Ga-fibroblast activation protein inhibitor PET/CT on gross tumour volume delineation for radiotherapy planning of oesophageal cancer. *Radiotherapy & Oncology*.

[41] Giesel F. L., Kratochwil C., Schlittenhardt J. (2021). Head-to-head intra-individual comparison of biodistribution and tumor uptake of 68Ga-FAPI and 18F-FDG PET/CT in cancer patients. *European Journal of Nuclear Medicine and Molecular Imaging*.

[42] Sollini M., Kirienko M., Gelardi F., Fiz F., Gozzi N., Chiti A. (2021). State-of-the-art of FAPI-PET imaging: a systematic review and meta-analysis. *European Journal of Nuclear Medicine and Molecular Imaging*.

[43] Zhou Y., Yang X., Liu H. (2021). Value of [68Ga]Ga-FAPI-04 imaging in the diagnosis of renal fibrosis. *European Journal of Nuclear Medicine and Molecular Imaging*.

[44] Finke D., Heckmann M. B., Herpel E. (2021). Early detection of checkpoint inhibitor-associated myocarditis using 68Ga-FAPI PET/CT. *Frontiers in cardiovascular medicine*.

[45] Siebermair J., Köhler M. I., Kupusovic J. (2020). Cardiac fibroblast activation detected by Ga-68 FAPI PET imaging as a potential novel biomarker of cardiac injury/remodeling. *Journal of Nuclear Cardiology*.

[46] Schmidkonz C., Rauber S., Atzinger A. (2020). Disentangling inflammatory from fibrotic disease activity by fibroblast activation protein imaging. *Annals of the Rheumatic Diseases*.

[47] Heckmann M. B., Reinhardt F., Finke D. (2020). Relationship between cardiac fibroblast activation protein activity by positron emission tomography and cardiovascular disease. *Circulation: Cardiovascular Imaging*.

[48] Zhang X., Song W., Qin C., Liu F., Lan X. (2021). Non-malignant findings of focal 68Ga-FAPI-04 uptake in pancreas. *European Journal of Nuclear Medicine and Molecular Imaging*.

[49] Pan Q., Luo Y., Zhang W. (2020). Idiopathic retroperitoneal fibrosis with intense uptake of 68Ga-fibroblast activation protein inhibitor and 18F-fdg. *Clinical Nuclear Medicine*.

[50] Song Y., Qin C, Liu F, Lan X (2021). Fibrous dysplasia mimicking skeletal metastasis on 68Ga-FAPI PET imaging. *Clinical Nuclear Medicine*.

[51] Hayrapetian A., Girgis M. D., Yanagawa J. (2020). Incidental detection of elastofibroma dorsi with 68Ga-FAPI-46 and 18F-fdg PET/CT in a patient with esophageal cancer. *Clinical Nuclear Medicine*.

[52] Zhang A., Zhang H., Zhou X., Li Z., Li N. (2021). Solitary fibrous tumors of the pleura shown on 18F-fdg and 68Ga-DOTA-FAPI-04 PET/CT. *Clinical Nuclear Medicine*.

[53] Windisch P., Zwahlen D. R., Giesel F. L. (2021). Clinical results of fibroblast activation protein (FAP) specific PET for non-malignant indications: systematic review. *EJNMMI Research*.

[54] Zhao L., Gu J., Fu K., Lin Q., Chen H. (2020). 68Ga-FAPI PET/CT in assessment of liver nodules in a cirrhotic patient. *Clinical Nuclear Medicine*.

[55] Liu H., Chen Z, Yang X, Fu W, Chen Y (2021). Increased 68Ga-FAPI uptake in chronic cholecystitis and degenerative osteophyte. *Clinical Nuclear Medicine*.

[56] Gu B., Luo Z., He X., Wang J., Song S. (2020). 68Ga-FAPI and 18F-fdg PET/CT images in a patient with extrapulmonary tuberculosis mimicking malignant tumor. *Clinical Nuclear Medicine*.

[57] Zhou Y., He J., Chen Y. (2021). 68Ga-FAPI PET/CT imaging in a patient with thyroiditis. *Endocrine*.

[58] Can C., Gã¼ndoÄŸan C, Gã¼zel Y, Kaplan Ä°, Kã¶mek H (2021). 68Ga-FAPI uptake of thyroiditis in a patient with breast cancer. *Clinical Nuclear Medicine*.

[59] Hao B., Wu X., Pang Y. (2021). [18F]FDG and [68Ga]Ga-DOTA-FAPI-04 PET/CT in the evaluation of tuberculous lesions. *European Journal of Nuclear Medicine and Molecular Imaging*.

[60] Luo Y., Pan Q., Zhang W., Li F. (2020). Intense FAPI uptake in inflammation may mask the tumor activity of pancreatic cancer in 68Ga-FAPI PET/CT. *Clinical Nuclear Medicine*.

[61] Wu J., Liu H., Ou L., Jiang G., Zhang C. (2021). FAPI uptake in a vertebral body fracture in a patient with lung cancer. *Clinical Nuclear Medicine*.

[62] Lin R., Lin Z., Zhang J., Yao S., Miao W. (2021). Increased 68Ga-FAPI-04 uptake in schmorl node in a patient with gastric cancer. *Clinical Nuclear Medicine*.

[63] Xu T., Zhao Y., Ding H. (2021). [68Ga]Ga-DOTA-FAPI-04 PET/CT imaging in a case of prostate cancer with shoulder arthritis. *European Journal of Nuclear Medicine and Molecular Imaging*.

[64] Qin C., Gai Y., Liu Q., Shao F., Lan X. (2020). Elevated 68Ga-FAPI accumulation in a recurrent angiomyolipoma. *Clinical Nuclear Medicine*.

[65] Liu H., Wang Y, Zhang W, Cai L, Chen Y (2021). Elevated 68Ga-FAPI activity in splenic hemangioma and pneumonia. *Clinical Nuclear Medicine*.

[66] Dendl K., Koerber S. A., Adeberg S. (2021). Physiological FAP-activation in a postpartum woman observed in oncological FAPI-PET/CT. *European Journal of Nuclear Medicine and Molecular Imaging*.

[67] Sonni I., Lee-Felker S., Memarzadeh S. (2021). 68Ga-FAPi-46 diffuse bilateral breast uptake in a patient with cervical cancer after hormonal stimulation. *European Journal of Nuclear Medicine and Molecular Imaging*.

[68] Wang L.-J., Zhang Y., Wu H.-B. (2020). Intense diffuse uptake of 68Ga-FAPI-04 in the breasts found by PET/CT in a patient with advanced nasopharyngeal carcinoma. *Clinical Nuclear Medicine*.

[69] Gündoğan C., Güzel Y., Can C., Alabalik U., Kömek H. (2021). False-positive 68Ga-fibroblast activation protein-specific inhibitor uptake of benign lymphoid tissue in a patient with breast cancer. *Clinical Nuclear Medicine*.

[70] Lindner T., Altmann A., Krämer S. (2020). Design and development of 99mTc-labeled FAPI tracers for SPECT imaging and 188Re therapy. *Journal of Nuclear Medicine*.

[71] Kuyumcu S., Kovan B, Sanli Y (2021). Safety of fibroblast activation protein-targeted radionuclide therapy by a low-dose dosimetric approach using 177Lu-FAPI04. *Clinical Nuclear Medicine*.

[72] Baum R. (2020). <strong>Peptide-Targeted radionuclide therapy (PTRT) using Lu-177 FAP-2286 in diverse adenocarcinomas: feasibility, biodistribution and preliminary dosimetry in a first-in-human study</strong>. *Journal of Nuclear Medicine*.

[73] Baum R. P., Schuchardt C., Singh A. (2021). Feasibility, biodistribution and preliminary dosimetry in peptide-targeted radionuclide therapy (PTRT) of diverse adenocarcinomas using 177Lu-FAP-2286: first-in-human results. *Journal of Nuclear Medicine*.

[74] Röhrich M., Syed M., Liew D. P. (2021). 68Ga-FAPI-PET/CT improves diagnostic staging and radiotherapy planning of adenoid cystic carcinomas - imaging analysis and histological validation. *Radiotherapy & Oncology*.

[75] Liermann J., Syed M., Ben-Josef E. (2021). Impact of FAPI-PET/CT on target volume definition in radiation therapy of locally recurrent pancreatic cancer. *Cancers*.

[76] Windisch P., Zwahlen D. R., Koerber S. A. (2020). Clinical results of fibroblast activation protein (FAP) specific PET and implications for radiotherapy planning: systematic review. *Cancers*.

