# Supplementary material for: Could Fibroblast Activation Protein (FAP)-Specific Radioligands Be Considered as Pan-Tumor Agents?
Source: Contrast Media Mol Imaging. 2022 Feb 22;2022:3948873. doi: 10.1155/2022/3948873 (PMC8888077; doi:10.1155/2022/3948873)
Supplement: Supplementary Materials — Supplementary Table 1: summary of clinical studies on FAP-tracers. Supplementary Table 2: characteristics of FAP-tracers. Supplementary Table 3: summary of case reports on FAP-tracers. [file 3948873.f1.zip › 3948873.f1/Supplementary table 1 (1) (1).docx]

Table 1. Summary of clinical studies on FAPI.

| **First author/**  **Year (Reference)** | | **Radiotracer**  **(dose) [n]** | **P.i. (minute)/Mode** | **Sample size (n)** | | **Study design**  **/Objective** | **Population study (n)** | **Quantitative/ Semi-quantitative parameters** | **Main finding(s)** | |
| --- | --- | --- | --- | --- | --- | --- | --- | --- | --- | --- |
| 1. **Oncologic Applications** | | | | | | | | | |  |
| [Bal](http://jnm.snmjournals.org/content/61/supplement_1/1013.short)/  2020 (1) ¶ | | [^68^Ga] Ga-DATA-FAPI-05  [^18^F] FDG | 60/3D (WB) | 12 | Case series /Biodistribution, dosimetry, and evaluation of FAPI PET uptake in Pts with inconclusive FDG PET results ∂ | | Different kind of cancers ¶ | SUV_max,_ SUV_mean_, and TBR values;  Dosimetry values | Potentially proper diagnostic value:  Higher mean TBR in half of the patients compared to FDG (9.9 Vs 7.2) and mean SUV_max_ of 12  possible theranostics application | |
| [Ballal](https://pubmed.ncbi.nlm.nih.gov/33244617/)/2020 (2) | | [^68^Ga] Ga-DOTA-SA-FAPI (185-370 MBq)  [^18^F] FDG (59.2-296 MBq) | 10,60 &180/3D (WB) [3]  60/3D (WB) [51] | 54 | Prospective study/ Evaluate the biodistribution, pharmacokinetics, dosimetry, and diagnostic value of DOTA.SA.FAPi compared to FDG in 14 kind of cancer | | Breast (20), Lung (13), H &N (4), GI (7), GU (3), myeloproliferative (3), Neuroblastoma (1), GBM (1), Neurofibroblastoma (1), Unknown primary (1) | SUL _Peak_ and SUL_Avg,_  Dosimetry values | Good biodistribution and dosimetry characteristics in studied cancers:  Effective dose: 1.11E-02 mSv/MBq similar to other FAPI-ligands  Critical organ: pancreas 5.46E-02 mGy/MBq  Imaging can acquire even 10 minutes post-injection.  Regarding patient-based comparison, complete FAPI concordance was observed in median SUL values of primary and second primary (4.4 vs. 6.1, *p*: 0.843) as well as metastases, except:  LN (concordance:92.5%, SULpeak 3.7 vs. 5, *p*: 0.672)  Lung nodules (concordance: 94.4%, SULpeak 5.7 vs. 5.4, *p*:0.375)  and brain metastases (concordance:92.5%, SULpeak 10.1 vs. 7.7, *p*: 0.477)  highest FAPI activity observed in H &N carcinomas and gallbladder cancers (SUL > 10)  Beside brain metastases, the other lesions revealed comparable SUL with not statistically significant differences between both tracers | |
| Baum/  2021 (3) | | [^177^Lu] Lu-DOTA-FAP 2286 (2.5-9.9 GBq)  [^68^Ga] Ga-FAP-2286 | SPECT/CT: 0.5, 2-3 h, 1, 2, 3 & 10 days P.i  PET/CT: N/A | 11 | Retrospective case series/ PTRT | | Metastatic adenocarcinoma:  Pancreatic (5), Breast (4), Ovarian (1) and colorectal (1) | SUV_max_ | whole-body effective dose for [^177^Lu] Lu-DOTA-FAP 2286: 0.07±0.02 Gy/GBq  Significant uptake in primary and metastatic lesions  with long retention of 177Lu-FAP-2286 according to SPECT/CT images performed 72 h to 10 days P.i  without grade 4 adverse events, but grade 3 adverse events in 3 Pts  Proper characteristics for theranostics application (favorable tumor retention time, mild adverse effects, desirable findings in post-treatment imaging): TBR>3 | |
| [Chen](https://www.researchgate.net/profile/Haojun_Chen/publication/340260494_Comparison_of_68GaGa-DOTA-FAPI-04_and_18F_FDG_PETCT_for_the_diagnosis_of_primary_and_metastatic_lesions_in_patients_with_various_types_of_cancer/links/5e834efb299bf130796c7e8c/Comparison-of-68GaGa-DOTA-FAPI-04-and-18F-FDG-PET-CT-for-the-diagnosis-of-primary-and-metastatic-lesions-in-patients-with-various-types-of-cancer.pdf)/  2020 (4) | | [^68^Ga] Ga-DOTA-FAPI-04 (1.8–2.2 MBq/kg)  [^18^F] FDG (3.7 MBq/kg) | 60/3D (WB) | 75 | Prospective cohort/ FAPI vs. FDG for evaluation of the primary source for CUP, as well as initial staging and recurrence evaluation and Diagnostic accuracy study | | 12 kind of cancers:  Lung (14), Liver (11), Nasopharyngeal (7), Gastric (8), Pancreatic (4), Esophageal (5), Glioma (4), Ovarian (6), Colorectal (8), Cervical (3), Sarcoma (3), Breast (1), Neuroendocrine tumor (3) § | SUV_max_, SUV_mean_, TBR values; | 68Ga-FAPI-04 uptake with a meanSUV of more than 10 in lung, CRC, cervical, pancreatic, gastric, liver, ovarian, nasopharyngeal carcinoma, esophageal and sarcoma  FAPI uptake significantly greater than FDG in most of the cases  Significantly higher FAPI sensitivity in detection rate of primary (98.2% vs. 82.1%, *P*: 0.021) and metastatic lesions: LNs (86.4% vs. 45.5%, *P*:0.004), bone and visceral metastases (83.8% vs. 59.5%, *P:* 0.004) as compared to that of FDG PET  Ga-FAPI PET had superior accuracy in detection of lesions in the brain, axial skeleton and liver as well as peritoneal carcinomatosis | |
| [Chen](https://www.researchgate.net/profile/Haojun_Chen/publication/342455476_Usefulness_of_68GaGa-DOTA-FAPI-04_PETCT_in_patients_presenting_with_inconclusive_18FFDG_PETCT_findings/links/5ef716fb299bf18816ea7e27/Usefulness-of-68GaGa-DOTA-FAPI-04-PET-CT-in-patients-presenting-with-inconclusive-18FFDG-PET-CT-findings.pdf)/  2020 (5) | | [^68^Ga] Ga-DOTA-FAPI-04 (1.8–2.2 MBq/kg)  [^18^F] FDG (3.7 MBq/kg) | 60/3D (WB) | 68 | Prospective Cohort/ Evaluating FAPI PET uptake in Pts with inconclusive FDG PET results ∂ | | Malignant (59):  GI (18), Lung (11), Hepatobiliary (13), H&N (7), Breast (4), GU (4), CUP (2)  Benign lesions (9):  Hepatic (3), TB (2), Pancreatic (2) and Esophagus (2) | SUV_max_, SUV_mean_, and TBR values | Statistically superior FAPI median SUV_max_ in primary lesions mainly hepatic malignancy (3.45vs. 12.29, *p*:0.03) and gastric carcinoma (2.70vs. 14.05, *p*:0.04)  Compared to FDG PET, significant higher uptake (median SUV_max_) was observed mainly in the peritoneal carcinomatosis (2.98vs. 10.14, *p*:0.005), liver (2.61vs. 9.28, *p*:0.008), and skeletal (1.71vs. 9.00, *p*:0.018) metastases  Significant superior lesion detection rate in FAPI PET in contrast to inconclusive FDG, prominently in gastric (57 vs. 15, *p*: 0.001), lung (20 vs. 5, *p*: 0.011), liver (19 vs. 06, *p*: 0.006), and NPC (16 vs. 08, *p*: 0.039) cancers  FAPI could serve as a complementary to FDG PET scan | |
| Dendl/  2021 (6) | | [^68^Ga] Ga-FAPI-02 (52-325MBq) [2]  [^68^Ga] Ga-FAPI-04 (52-325MBq) [17]  [^68^Ga] Ga-FAPI-46 (52-325MBq) [12]  [^18^F] FDG (251-300 MBq) [10] | 60/3D (WB) | 31 | Retrospective Study/biodistribution, gynecological tumor, and hormone sensitive organs Ga-FAPI uptake | | Several gynecological tumors:  Breast cancer; ovarian cancer; cervical cancer; endometrial cancer; leiomyosarcoma of the uterus; tubal cancer | SUV_max_, SUV_mean_, and TBR values | Ga-FAPI PET/CT showed a mean  SUVmax of 10.3 (range 4.6–14.4) for primary tumors and  12.3 (range 5.6–21.6) for local relapses, as well as 10.0  (range 3.9–29.0) for metastases  The highest uptake belonged  to endometrial carcinoma with a mean SUVmax of 18.4  Significantly higher mean SUVmax in premenopausal vs. postmenopausal women in endometrium (11.7 vs. 3.0; *p*< 0.001) and breast (1.8 vs. 1.0  *p*=0.004). | |
| [Ferdinandus](https://pubmed.ncbi.nlm.nih.gov/33620560/)/ 2021 (7) | | [^68^Ga] Ga- FAPI-46  (148 MBq) | 10,60/3D (WB) | 69 | Retrospective Study/ Investigate the proper time-point for imaging regarding early(10-min) or late (60-min) post injection in different type of cancers | | Restaging (52), Staging (17):  Sarcoma (33), GI &Hepatobiliary (21), GU (5), Thyroid (2), Mesothelioma (1) and NSCLC (7) | SUV_max_, SUV_mean_ and TBR values | No significant changes in SUV_max_ between early and late imaging in basis of per-lesion and per-patient analysis in Primary (median SUVmax lesion 12.0 vs. 14.2, *p*:0.33, respectively) and metastatic lesions (e.g., LNs 9.15vs. 11.2, *p*:0.14)  Significant(*p*<0.001) decremental early to late proportion in SUV_mean_ of gluteus and liver as well as mediastinum, respectively  Early time-point is preferable for Ga-FAPI-46 PET scan | |
| [Geist](https://www.researchsquare.com/article/rs-16740/latest.pdf)/  2020 (8) | | [^68^Ga] Ga-FAPI-04 | Up to 60 /Dynamic | 8 | Five dynamic models studied to discriminate HCC from other hepatic lesions Ω | | HCC (4), ICC (2), Liver metastases from GI (1), Inflammatory granulomatous (1) | SUV_max_, SUV_mean_;  Kinetic models | Dynamic FAPI PET using two-tissue compartment model could show significant differences in the kinetic parameters between HCC, non-HCC lesions, and healthy liver parenchyma (P<0.01) | |
| Giesel/  2021 (9) | | [^68^Ga] Ga-FAPI-02(52-325 MBq) [6]  [^68^Ga] Ga-FAPI-04(52-325 MBq) [32]  [^68^Ga] Ga-FAPI-46(52-325 MBq) [32]  [^68^Ga] Ga-FAPI-74 (52-325 MBq) [1]  [^18^F] FDG (233-680 MBq) | 60/3D (WB) | 71 | Prosospective Study/ Biodistribution & comparison of tumor uptake of Ga-FAPI and FDG radiotracers | | Different cancers:  H&N (16), lung (9), pancreato-biliary (12), GI (14), gynecologic (12) & other cancers (8) | SUV_max_, SUV_mean_, and TBR values | No significant difference in mean SUV_max_ values of [^68^Ga] Ga-FAPI vs. [^18^F] FDG in primary tumors (12.14 vs. 11.69; *p*=0.429) & metastatic lesions (8.49 vs. 9.48; *p*=0.814)  TBRs of LN & lung metastases were similar between [^68^Ga] Ga-FAPI & [^18^F] FDG (17.4 & 24.8, respectively; *p*=0.132), whilst TBRs of bone & hepatic metastases were significantly higher in [^68^Ga] Ga-FAPI vs. [^18^F] FDG (7.2 vs. 3.3;  p=0.033 & 5.8 vs. 2.6; p=0.011) | |
| [Giesel](http://jnm.snmjournals.org/content/early/2020/06/26/jnumed.120.245084.full.pdf)/  2020 (10) | | [^68^Ga] Ga-NOTA-FAPI-74 (263 MBq) (cold kit) [1]  [^18^F] F-AIF-NOTA-FAPI-74 (259±26 MBq) [9] | 10,60 &180/3D | 10 | Retrospective Study/Biodistribution and Dosimetry. Also, assessment of FAPI in tumor delineation (central-located pulmonary lesions) | | Lung cancer:  Adenocarcinoma (8), SCC (2) | SUV_max_, SUV_mean_, and TBR values;  Dosimetry values and GTV | Effective dose for  [^18^F] F-FAPI-74 calculated 1.4±0.2 mSv/100 MBq (lower than FDG values) and for [68Ga] Ga-FAPI-74 was 1.6 mSv/ per 100 MBq  Critical organ: urinary bladder  3,41 mSv/100MBq for [18F] F-FAPI-74 and 4.66 mSv/100MBq for [68Ga] Ga-FAPI-74  Favorable potential utility for theranostics purposes  One-hour p.i is favorable time-point despite minimally reduced tumor retention-time (SUV_max_>10 in primary, LNs and metastatic lesions)  Blood pool activity slightly higher than other FAPI-conjugated radioligands (owing to vessel visualization)  Sufficient AlF-FAPI PET-based tumor delineation as compare to CT and FDG PET (considering PET-GTV threshold x3) η | |
| [Giesel](http://jnm.snmjournals.org/content/60/3/386.full.pdf)/  2019 (11) | | [^68^Ga] Ga-DOTA-FAPI-02 (122–336 MBq) [25]  [^68^Ga] Ga-DOTA-FAPI-04 (122–336 MBq) [25]  [^18^F] FDG [6] | 60/3D (WB) | 50 | Prospective cohort/ Biodistribution and dosimetry | | Breast (2), H&N (8), CUP (2), CRC (4), HCC (2), Liposarcoma (1), Pancreatic (13), Prostate (4), RCC (1), Thyroid (4), NSCLC (5), Esophageal (2), Uterus (1), Ovarian (1) | SUV_max_, SUV_mean_, and TBR values | Proper dosimetry for both FAPI-02& FAPI-04 (Effective dose 1.80E−02 & 1.64E−02 mSv/MBq, respectively)  Favorable biodistribution:  SUV_max_ of 8.37 vs. 10.07 (FAPI-02 vs. FAPI-04) with no significant difference and same TBR after one-hour p.i  High uptake with similar quantitative values of 68Ga-FAPI-02/04 & 18F-FDG (average SUVmax of 7.37 & 7.41) prominently in the pancreatic, esophageal, NSCLC, CRC, and H&N cancers  FAPI imaging showed better visualization and lower background activity in the brain, liver, and oral–laryngeal mucosa in contrast to FDG PET imaging (diagnostic accuracy study was not performed) | |
| [Guo](https://pubmed.ncbi.nlm.nih.gov/33179149/)/ 2020 (12) | | [^68^Ga] Ga-DOTA-FAPI-04 (148-259 MBq)  [^18^F] FDG (3.7 MBq/kg) | 60/3D (WB) | 34 | Retrospective study/FAPI diagnostic utility for detection of primary and metastatic liver lesions compare to anatomic modalities and FDG PET | | Indefinite hepatic lesions:  HCC (20), ICC (12) and benign nodule (2) | SUV_max_ and TBR values | Sensitivity of FAPI for detection of intra-hepatic (primary)lesions was non-significantly lower than CE-CT & MRI (96% vs. 96%&100%, *P*>0.99), while statistically superior to FDG (96% vs. 65%, *P* < 0.001)  Concerning detection of all lesions, FAPI was seen significantly higher than FDG (87.4% vs. 65%, *P*<0.001)  Regarding metastasis evaluation, FAPI revealed more detection rate of metastatic LNs, bone metastases and peritoneal carcinomatosis compared to FDG (except lung metastases: 9 vs. 18 lesions)  Median SUV_max_ values of FAPI were significantly higher than FDG for primary lesions (13.61 vs. 4.24; *P* < 0.001, TBR 22 vs. 15 *P* < 0.001), and metastases (6.72-9.21 vs. 2.62-2.88), except lungs metastases (1.58 vs. 1.42, *p*: 0.294)  Significant correlation between the 68Ga-FAPI-04 derived SUVmax and TBR with pathologic grades of HCC (*p*<0.05)  Benign lesions showed no remarkable uptake in FAPI PET | |
| Jin / 2021 (13) | | [^68^Ga] Ga-FAPI-04 (1.8–2.2 MBq/kg) | 60 (WB) | 73 | Prospective study/ FAPI diagnostic utility for detection of fibroblast activation proteins in lymphoma | | Lymphoma:  HL (11), NHL (62) | SUV_max_ and sum of  the product of the diameters | The average SUVmax and SUVmax  range of the lymphoma lesions were 9.46±4.61, & 1.7-23.3, respectively  The highest average SUVmax (>10) belonged to primary mediastinal large BCL, Burkitt lymphoma, HL, and DLBCL; whilst the  lowest (<5) belonged to MALT lymphoma  No statistically significant correlation observed between the sum of the product of the diameters and corresponding SUVmax | |
| Kessler/  2021 (14) | | [^68^Ga] Ga-FAPI-46 (144±36 MBq) [47]  [^18^F] FDG (214±102 MBq) [43] | 10 ([^68^Ga] Ga-FAPI-46)  60 ([^18^F] FDG) | 47 | Prospective study/evaluation of association between the intensity of 68Ga-FAPI uptake and histopathological FAP expression | | Bone or soft tissue sarcomas | SUV_max_ | Patient based detection rates for FAPI and FDG PET scans were 76.6% and 81.4%, respectively  Association between intensity of FAPI-PET uptake and  histopathological FAP-expression was significant  Compared to FDG-PET, FAPI-PET resulted in upstaging in 18.6% of Pts and clinical management changes 30% of Pts | |
| Koerber/  2021 (15) | | [^68^Ga] Ga-FAPI-04  (127-308 MBq) [6]  [^68^Ga] Ga-FAPI-46 (127-308 MBq) [5]  [^68^Ga] Ga-FAPI-74 (127-308 MBq) [4] | 60/3D | 15 | Retrospective study/ Imaging and tumor delineation of sarcoma using Ga-FAPI PET/CT scan | | Different types of sarcomas:  Liposarcomas (5), undifferentiated pleomorphic  sarcomas (3), leiomyosarcomas (2) & others (5) | SUV_max_, SUV_mean_, and TBR values | High median SUV_max_ values in primary tumors (7.16), local recurrence (11.47), & metastases (6.29); as well as excellent TBRs (>7)  SUV_max_ values more than 10 was reported for more aggressive diseases | |
| [Koerber](http://jnm.snmjournals.org/content/early/2020/02/13/jnumed.119.237016.full.pdf)/  2020 (16) | | [^68^Ga] Ga-FAPI-04 (111 to 298 MBq) [16]  [^68^Ga] Ga-FAPI-46 (111-298 MBq) [6] | 60/3D (WB) | 22 | Retrospective cohort/ Imaging of lower GI malignancies with FAPI and compare with conventional anatomical imaging | | Lower GI tract malignancies:  Treatment-naïve anal cancer (6), Metastatic lower GI cancers (16) | SUV_max_, SUV_mean_, and TBR values | Favorable uptake with both tracers (overall primary tumoral and metastases SUV_max_: 12.59 ± 7.46 and 7.95± 3.49, respectively) and TBR (>3, *p*<0.002)  The highest uptake seen in anal cancer and hepatic metastases (SUV_max_ 13.9 and 9.1± 3.6)  Changing in TNM staging in nearly half of  primary/metastatic Pts (50% & 47%, respectively).  Changes in the oncological management of either primary/metastatic Pts were reported in 81% of cases | |
| Komek/  2021 (17) | | [^68^Ga] Ga-FAPI-04 (2 MBq/kg)  [^18^F] FDG (3.5-5.5 MBq/kg) | 60/3D (WB) | 20 | Prospective pilot study/ Imaging of breast malignant tumors with FAPI and compare with FDG | | Primary and recurrent breast cancer | SUV_max_, and TBR values | The FAPI derived mean SUVmax were significantly higher than FDG derived values for primary tumors (16.4 vs. 71; *p*<0.001), lymph nodes (15.4 vs. 4.3; *p*=0.003), lung metastases (7.5 vs. 3.3; *p*=0.006), and bone metastases (7.3 vs. 4.9; *p*<0.001), except liver metastases (9.9 vs. 7.1; *p*>0.05)  FAPI PET/CT showed sensitivity and specificity of 100% and 95.6%, respectively in detecting primary breast lesions, while the sensitivity and specificity of FDG PET/CT were 78.2% and 100%, respectively | |
| [Kratochwil](http://jnm.snmjournals.org/content/60/6/801.full.pdf)/  2019 (18) | | [^68^Ga] Ga-DOTA-FAPI-04 (122–312 MBq) | 60/3D (WB) | 80 | Prospective cohort/Evaluating FAPI uptake in 28 different kinds of challenging malignancies with equivocal CI results | | Primary tumor (54) & Metastatic lesions (229):  CUP (7), sarcoma (8), GI & Hepatobiliary (128), Breast (12), Lung (25), GU (12), H&N (44), Prostate (16), Desmoid (4), Chordoma (1), MTC (6), ACC (4), Pheochromocytoma (4) and DTC (12) | SUV_max_, SUV_mean_, and TBR values | Remarkable uptake (SUV_max_ >12 & TBR>6) mainly in CUP, Sarcoma, Breast, salivary gland, esophageal, cholangiocarcinoma, breast, and lung cancers  Intermediate uptake (SUV_max_ >6 & TBR>3) in prostate, pancreatic, thymus, H&N, ovarian, desmoid tumors, chordoma, colorectal, anal, NET, small intestine, MTC, cervical cancer and HCC  Lowest SUV_max_ (<6) were acquired in RCC, DTC, Adenoid cystic, Pheochromocytoma, insulinoma, gastric and neuroendocrine prostate cancer  Neither SUV_mean_ (11.5 ± 5.5 vs. 10.0 ± 6.3) nor SUV range (2.9-21.6 vs. 2.0-44.8) of 68Ga-FAPI-04 uptake in primary tumors and metastatic lesions significantly differ  Similar to FDG PET, cases with DTC and RCC showed low uptake in FAPI PET imaging | |
| [Kuyumcu](https://pubmed.ncbi.nlm.nih.gov/33883494/)/ 2021 (19) | | [177 Lu] Lu-DOTA-FAPI-04 (267.5 ± 8.6 MBq) | 4,24,48 &96 Hrs/WB & 3D SPECT/CT | 4 | Prospective (Dosimetry) Cohort/End-stage refractory metastatic cases with Ga-FAPI avidity were evaluated for prospect of low-dose theranostics approach | | Refractory Advanced Metastatic Cancers:  Triple-Neg Breast (1), Thymic carcinoma (1), PTC (1) and Ovarian carcinosarcoma (1) | SUV_max_, SUV_mean_ and Dosimetry values | Reasonable and low critical-organ absorbed dose (mean bone marrow–absorbed dose 0.04 mGy/MBq) as opposed to other conventional Peptide radio-ligand theranostics, e.g., [177 Lu] Lu-PSMA-617 and [177 Lu] Lu-DOTATATE  While tolerated dose of bone marrow estimated up to 50 GBq, absorbed-dose of malignant lesions were low  Tumoral uptake was similar to Ga-FAPI-04 results  Bone involvement absorbed the highest dose (0.62 ± 0.55 mGy/MBq) subsequent to LN (0.38 ± 0.22 mGy/MBq) and hepatic (0.33 ± 0.21 mGy/MBq) metastases | |
| [Liermann](https://pubmed.ncbi.nlm.nih.gov/33672893/)/  2021(20) | | [^68^Ga] Ga- FAPI-04 (1.8-2.2 MBq/kg) | 40-60/3D (WB) | 7 | Retrospective Study/FAPI PET-based GTV contouring in comparison with conventional CT-based tumor delineation | | Locally recurrent Pancreatic malignancies:  Ductal adenocarcinoma (7) | SUV_max_, TBR values, and GTV | High FAPI uptake and TBR in pancreatic lesions, suggest superiority to conventional anatomic imaging  Although inter-observer variability differed significantly (*p*<0.05), FAPI-based x2 threshold GTV (median 21.0 ccm) showed precise delineation with no significant difference as compare to manual (median between 15.8 and 42.3 ccm) conventional radiotherapy-plan mapping | |
| [Lindner](https://pubmed.ncbi.nlm.nih.gov/32169911/)/2020 (21) | | [^99m^Tc] Tc-FAPI‐34 (660 MBq)  [^90^ Y] Y-FAPI-46 (6 GBq) | 10,60,240 & 1200 (20Hrs)/ WB & 3D SPECT/CT | 2 | Part of a preclinical study (proof-of-concept)/ uptake evaluation and compare with Ga-FAPI PET | | Advanced Metastatic Pts:  Ovarian (1), Pancreatic (1) | N/A | Demonstrated that Tc-FAPI-34 could be a proper SPECT tracer and considering DOTA chelator, would be suitable for theranostics application via Re-188  Each patient received 6GBq radiolabeled yttrium after obtaining eligibility with Ga-FAPI-46  6- to 8-week follow-up period with Tc-FAPI‐34 | |
| Linz/  2021 (22) | | [^68^Ga] Ga- FAPI-04 (66-168 MBq)  [^18^F] FDG (204-317 MBq) | 60/3D (WB) | 10 | Prospective pilot study/ uptake evaluation and compare with [^18^F] FDG PET/CT & MRI | | Treatment naïve oral SCC | SUV _max_, SUV_peak_, | FAP specific PET/CT & FDG PET/CT demonstrated  comparable sensitivity (81.3% vs. 87.5%; *p* = 0.32) and specificity  (93.3% vs. 81.3%; *p* = 0.16) in detection of cervical LN metastases  [^18^F] FDG and [^68^Ga] Ga- FAPI-04 PET/CT identified all primary tumors with SUVmax value of 25.5±13.2 & 20.5±6.4, respectively | |
| [Meyer](http://jnm.snmjournals.org/content/61/8/1171.short)/  2019 (23) | | [^68^Ga] Ga-FAPI-46 (214-246 MBq) | 10,60 &180/3D | 6 | Retrospective study/Biodistribution and Dosimetry | | Advanced GI (2), H&N (2), Breast (1), Cholangiocellular carcinoma (1) | SUV _max_, SUV_mean_, and TBR values;  Dosimetry values | Favorable dosimetry and kinetic profile (especially prolonged tumoral retention) support therapeutic applications  Average effective total body dose: (7.80E-03 mSv/MBq)  Critical organ: Bladder wall (2.41E-03 mSv/MBq) | |
| [Pang](https://pubmed.ncbi.nlm.nih.gov/33258746/) /2020 (24) | | [^68^Ga] Ga-DOTA-FAPI-04 (1.8-2.2 MBq/kg)  [^18^F] FDG (3.7 MBq/kg) | 60/3D (WB) | 35 | Retrospective study /Comparison with FDG with aim to evaluation diagnostic performance and changing TNM staging and treatment plan | | Upper &Lower GI Adenocarcinomas and Signet-ring cell carcinoma:  Gastric (20), Duodenal (2), and Colorectal (13) | SUV_max_ | Significant higher detection rate and FAPI uptake as compared to the FDG in the:  Primary involvement (100% vs. 53%, *p*: 0.004 & gastric: SUV_max_, 12.7 vs. 3.7, *P* :0.003, colorectal: SUV_max_, 12.7 vs. 3.7, *P* :0.003); LNs (79% vs. 54%, median SUV_max_, 6.7 vs. 2.4; *P*: 0.001); bone and visceral metastases (89% vs. 57%, *p*<0.001, SUV_max_ 2-3 times higher as compared to FDG)  Up-staging 4 out of 19 treatment-naïve Pts | |
| [Qin](https://pubmed.ncbi.nlm.nih.gov/33863819/)/ 2021 (25) | | [^68^Ga] Ga- FAPI-04 (1.85-3.7 MBq/kg)  [^18^F] FDG (3.7-5.55 MBq/kg) | 30-60/3D (WB) & MR | 20 | Prospective study /Lesion- and patient-based comparison of primary and metastatic detection rate of FAPI PET/MR with FDG PET/CT | | Gastric carcinoma | SUV_max,_ Normalized SUV_max,_ and T1WI, T2WI and DWI | Significantly higher 68Ga-FAPI-04 uptake in primary sites than that of FDG (SUV_max_ of 11.31±3.96 vs. 6.18±2.46; P<0.05)  FAPI revealed significantly superior at detection of primary site (100% vs. 71.43%, *p:*0.034) and metastatic lesions, in comparison to FDG; except ovarian (12 vs. 12 lesions) and supradiaphragmatic LN (18 vs. 24 lesions) metastases  FAPI can be consider as a substitution to FDG | |
| [Qin](https://pubmed.ncbi.nlm.nih.gov/33609152/)/2021  (26) | | [^68^Ga] Ga- DOTA-FAPI-04 (1.8-2.2 MBq/kg)  [^18^F] FDG (3.7-5.4 MBq/kg) | 30-60/3D (H&N)  PET-MR | 15 | Prospective Study/Diagnostic performance of FAPI PET/MR along with FDG PET/MR in NPC | | Recent-diagnosed Nasopharyngeal carcinomas (NPC):  well-diff. keratinizing SCC (3), nonkeratinizing diff. carcinoma (2), nonkeratinizing undiff. Carcinoma (8), keratinizing carcinoma with unknown diff. (1) and a recurrent NPC | SUV_max_, and GTV | In category of detection rate, both tracers revealed same sensitivity (both 100%)  Tumor delineation for radiotherapy, FAPI with threshold of 25% and FDG 20% of SUV_max_ showed the same accuracy as MRI (interclass correlation coefficient 0.85 to 0.91)  While FAPI have changed staging in 6 out of 15 (3up-stage and 3 down-stage), primary tumor uptake was non significantly lower than FDG (13.87 ± 5.13 vs. 17.73 ± 6.84, *p*: 0.078)  In case of LN detection, FAPI depicted less LN (48 vs. 100) with lower uptake; statistically significant as compare to FDG (11.94 ± 6.15 vs. 8.81 ± 3.79, *p*: 0.000)  FAPI outperformed FDG in diagnosing skull-base (7 vs. 0) and intracranial invasion (4 vs. 0 Pts) | |
| [Ristau](https://pubmed.ncbi.nlm.nih.gov/33063132/)/  2020 (27) | | [^68^Ga] Ga-FAPI-04 (180-325 MBq) [6]  [^68^Ga] Ga-FAPI-46 (180-325 MBq) [1] | 60/3D (WB) | 7 | Retrospective study/ Initial staging and tumor delineation for radiotherapy planning compared to Endo-esophageal clipping as a standard reference | | Newly-diagnosed esophageal malignancy:  SCC (6), Adenocarcinoma (1) | SUV_max_, SUV_mean_, and TBR values, and GTV | Satisfactory primary tumor (7/7 Pts median SUV_max_: 17.2) delineation and  metastatic LN (median SUV_max_: 9.7) detection more than CT images  Tumor delineation for radiotherapy altered in almost all cases (due to superb TBR more than 11) in comparison with conventional CT scan (median GTVs: 37.73 vs. 34.25 cm^3^) | |
| Röhrich/  2021 (28) | | [^68^Ga] Ga-FAPI-2 (150-250 MBq) [2]  [^68^Ga] Ga-FAPI-  46 (150-250 MBq) [7]  [^68^Ga] Ga-FAPI-74 (150-250 MBq) [3] | 10,60 &180/3D | 12 | Retrospective case series/ FAPI PET imaging in tumor delineation vs. MRI & CT parameters for radiotherapy planning | | Adenoid cystic carcinomas | SUV_max_, SUV_mean,_ TBR and GTV | Average SUV_max_ and SUV_mean_ values  of the primary lesions were 11.16±4.07 & 5.10±1.76, respectively; with TBRs  6.64±3.13 for SUV_max_ & 5.48±2.30 for SUV_mean_  Compared to the conventional methodologies, 68Ga-FAPI-PET/CT led to alteration in staging of  42% of Pts  In comparison to CT and MRI 68Ga-FAPI PET improved the accuracy of GTV delineation for radiotherapy planning | |
| [Röhrich](https://www.ncbi.nlm.nih.gov/pubmed/32344293)/  [2020](https://www.sciencedirect.com/science/article/pii/S2352340920306065) (29) | | [^68^Ga] Ga-DOTA-FAPI-02 (200 ± 50 MBq) [2]  [^68^Ga] Ga-DOTA-FAPI-04 (200 ± 50 MBq) [11] | 10,60 &180/3D [2]  30/3D (WB) [11] | 13 | Retrospective case series/FAPI PET imaging vs. MRI parameters; characterizing the clinical value of FAPI PET imaging in glioblastoma | | Confirmed IDH-wild type glioblastomas | SUV_max_, SUV_mean_, and TBR values;  MRI parameters (ADC, rCBV), and GTV | FAPI uptake indicated positive mild correlation with rCBV (r=0.385, *p*<0.0001); nevertheless, showed no association (r=0.061, *p*<0.0001) with cell density equivalent (ADC) | |
| [Röhrich](https://pubmed.ncbi.nlm.nih.gov/31388723/)/  2019 (30) | | [^68^Ga] Ga-DOTA-FAPI-02 (200 ± 50 MBq) [2]  [^68^Ga] Ga-DOTA-FAPI-04 (200 ± 50 MBq) [16] | 10,60 &180/3D (WB) [2]  30/3D (WB) [16] | 18 | Retrospective case series /Comparison of CI, IHC and FAP expression with FAPI imaging | | Histopathologically confirmed IDH-wildtype glioblastomas (14), grade II-IV IDH-mutant gliomas (4) | SUV_max_, SUV_mean_, and TBR values | High tracer uptake in the IDH-wildtype glioblastomas (average SUVmax 4.21 ±2.38) and IDH-mutant high-grade gliomas (III-IV) (SUVmax 3.4 and 2.2) compare to IDH-mutant glioma grade-II (0.35 ± 0.10)  FAPI may serve as an imaging modality for high-grade (III-IV) transformation of Gliomas | |
| [Rohrich](https://pubmed.ncbi.nlm.nih.gov/33097632/)/2020 (31) | | [^68^Ga] Ga-FAPI-04 (167-293 MBq) [16]  [^68^Ga] Ga-FAPI-46 (167-293 MBq) [3] | 10,60 &180/3D (WB) [6]  60/3D (WB) [13] | 19 | Retrospective study /Evaluation of management-plan modification of pancreatic ductal adenocarcinomas and compare to CE-CT | | Pancreatic Ductal Adenocarcinomas:  Primary (7), Metastatic (12) | SUV_max_ and SUV_mean_ | Primary tumor showed higher SUV_max_ than recurrence (17.41 ± 7.40 vs. 11.90 ± 3.31)  In addition, LN and distant metastases revealed high uptake value (14.13 ± 8.50 and 7.34 ± 2.48, respectively)  In contrast to pancreatitis, tumoral lesions depicted higher SUV_max_ value (15.64 ± 5.81 vs. 7.50 ± 3.52)  FAPI as compare to routine CE-CT, changed TNM staging in almost half of the patients (47% up-stage and 5% down-stage) | |
| [Serfling](https://pubmed.ncbi.nlm.nih.gov/33057927/)/  2020 (32) | | [^68^Ga] Ga-FAPI-04 (average 145 MBq)  [^18^F] FDG (292 ± 32 MBq) | 60/3D (WB) | 8 | Retrospective study/  Ga-FAPI vs FDG PET for Pre-op localization  HPV status obtained,  Histopathology and IHC served as gold standard | | Suspected Waldeyer’s tonsillar ring carcinoma or CUP:  Palatine (7) and Lingual (1) SCC | SUV_max_, SUV_mean_, and TBR values | While higher uptake was observed in primary tumor in FDG PET (21.29 vs.16.06, p:0.2), uptake ratio (relative to contralateral) was significantly greater in FAPI than FDG (4.47 vs. 2.68, *p*:0.03)  FAPI LN-detection rate reported 48% with no concordance with HPV positivity, although FDG revealing 82% | |
| [Shi](https://pubmed.ncbi.nlm.nih.gov/33097975/)/2020  (33) | | [^68^Ga] Ga-FAPI-04 (196-260 MBq)  [^18^F] FDG (3.7 MBq/kg) | 40-45/3D (WB) | 20 | Prospective study/ FAPI PET performance, comparison with FDG PET regarding to diagnostic value of hepatic tumors | | Unknown intrahepatic lesions:  HCC (16), ICC (4), Hemangiomas (2) and Granuloma (1) | SUV_max_, SUV_mean_, and TBR values, IHC from 7Pts | FAPI represented significantly higher detection rate compared to FDG in malignant primary hepatic lesions per patient (sensitivity 100% vs. 58.8% and specificity 100% vs. 100%)  and per lesion (sensitivity 100% vs. 55.0%, specificity 100% for both) point of view.  No significant different in uptake between cirrhotic and non-cirrhotic Pts.  Statistically significant higher FAPI SUV_max_ compare to FDG in HCC (8.47 ± 4.06 vs. 4.86 ± 3.58, *p*: 0.007) and ICC (14.14 ± 2.20 vs. 9.19 ± 3.60, *p*: 0.023) lesions  Considering mild FAPI uptake in benign lesions, it could be able to differentiated benign hepatic masses from malignant ones | |
| [Shi](https://link.springer.com/article/10.1007%2Fs00259-020-04882-z)/  2020 (34) | | [^68^Ga] Ga-FAPI-04 (96-260 MBq) | 60/3D (WB) | 17 | Prospective cohort/ correlating FAPI PET imaging findings with FAP expression by IHC | | Hepatic lesions suspected for malignancy:  HCC (11), ICC (2), metastases (3), benign granulation tissue (1) | SUV_max_, SUV_mean_, and TBR values;  IHC (score 0-4) | No significant correlation founded between Ga-FAPI uptake and FAP expression (IHC), probably due to non-uniform expression of FAP in HCCs  High sensitivity (100%) in detection of intrahepatic malignant lesions with statistically significant (*p*<0.05) different SUVs_max_ (HCC :7.78 ± 3.84, ICC:13.55 ± 2.34 and Metastases: 7.67 ± 4.56)  Within HCC lesions, poorly-diff. lesions revealed non-significant higher uptake  Non-tumoral liver uptake of cirrhotic patients (SUV mean= 1.39 ± 0.75) was significantly higher than non-cirrhotic cases (SUV mean= 0.46 ± 0.05) *p*<0.05.  Benign lesion revealed slight uptake | |
| [Syed](https://scholar.google.com/scholar?hl=en&as_sdt=0,5&q=Fibroblast+activation+protein+inhibitor+%28FAPI%29+PET+for+diagnostics+and+advanced+targeted+radiotherapy+in+head+and+neck+cancers&btnG=)/  2020 (35) | | [^68^Ga] Ga-FAPI (80 nmol/ GBq) ∅ | 30/3D (WB) | 14 | Prospective cohort/ FAPI imaging vs. CI modalities (CE-CT, MRI) for RT planning | | Complicated H&N cancers:  SCC (12), Mucoepidermoid carcinoma (1), Undifferentiated (1) | SUV_max_, SUV_mean_, TBR values;  Target volumetric parameters | High 68Ga-FAPI uptake in primary tumor (SUVmax: 14.62±4.44), lymph node (SUVmax: 9.42 ±5.72) and bone metastases (SUVmax: 7.51-14.62)  Multiple FAPI derived-GTVs significantly differ and unconcordant in tumor delineation as compare to CT-GTV (FAPI × 3, *p*:0.0134, FAPI × 5, *p*: 0.0419, FAPI × 7, *p*: 0.0001, FAPI × 10, *p*: 0.0001)  Combined CT-GTV with FAPI-GTV were significantly larger than CT-GTV in x3 and x5 threshold (*p* = 0.0005 and *p* = 0.0122, respectively)  Diagnostic accuracy of FAPI-based GTVs could not be validated, since no histopathological gold standard was available | |
| Wang/  2021 (36) | | [^18^F] AlF-NOTA-FAPI (173.5 to 256.8 MBq)  [^18^F] FDG | 60-90/ (WB) | 10 | Prospective study/bio distribution, dosimetery and tumor detection efficacy compared to the FDG | | Different cancer types:  lung cancer, pancreas cancer, colorectal cancer,  prostate cancer and lymphoma patients | SUV_max_, SUV_mean_, TBR values | The whole body effective dose:  1.24E−02 mSv/MBq  Critical organ: Osteogenic cells 2.47E−02 mGy/MBq | |
| [Wang](https://pubmed.ncbi.nlm.nih.gov/33777814/)/2021 (37) | | [^68^Ga] Ga- DOTA-FAPI-04 (103.2-257.5 MBq)  [^18^F] FDG (3.7-5.55 MBq/kg) | Up to 60 /Dynamic (WB) | 6 | Prospective Study/Evaluation of FAPI uptake in Chinese Healthy volunteer and lung cancer Pts in comparison with FDG | | Healthy volunteer (3), and Lung carcinoma (3) | SUL_max_, SUL_mean_, SUV_max_, SUV_mean_ and Dosimetry values | Whole-body effective dose (1.27E-02 vs. 1.64E-02) was relatively as same as the previous studies (Caucasian)  Critical organ: urinary bladder 1.45E-01 mGy/MBq  Evidence of proper diagnostic value in lung Pts  SUV _mean_ might be useful than SUL _mean_ in this study  Uptake in lung lesions increased over time during dynamic phase | |
| [Windisch](https://www.sciencedirect.com/science/article/pii/S0167814020303650)/  2020 (38) | | [^68^Ga] Ga-DOTA-FAPI-02 (200 ± 50 MBq) [2]  [^68^Ga] Ga-DOTA-FAPI-04 (200 ± 50 MBq) [11] | 30/3D (N/A) | 13 | Prospective study/  FAPI PET imaging for target volume delineation in glioblastoma and correlated to MRI | | Histologically conﬁrmed glioblastoma with residual tumor in the recent imaging | SUV_max_, SUV_mean_, and TBR values;  MRI-GTV, PET-GTV | Derived size (volume) of PET-GTVs in comparison with MRI-GTV were different significantly for FAPx5 threshold (*p* :0.022) while not for FAPx7 and FAPx10 (*p* :0.95 and 0.068, respectively).  Significant increase in added PET-GTVs (in all three thresholds) to MRI-derived GTV was reported (p < 0.001)  Tumor volume delineation based on FAPI PET, no signiﬁcantly correlated with survival. However, no complication was reported. | |
| [Zhao](https://pubmed.ncbi.nlm.nih.gov/33415432/)/2021 (39) | | [^68^Ga] Ga- DOTA-FAPI-04 (1.8-2.2 MBq/kg)  [^18^F] FDG (3.7 MBq/kg) | 60/3D (WB) | 46 | Retrospective Study/ Diagnostic(detection) application of FAPI in comparison with FDG toward various kind of malignancies with suspicious peritoneal involvement | | Lesion types:  Nodular-type peritoneal carcinomatosis (27), diffuse-type peritoneal carcinomatosis (16) and true-negative patients (3) | SUV_max_, and 13-region peritoneal cancer index (PCI) scoring system | FAPI observed with significant higher mean SUV_max_ (9.82 vs. 3.48 total, *P*<0.001) and sensitivity in detection (97.67% vs. 72.09% total; *P*: 0.002) of peritoneal carcinomatosis, especially in gastric malignancies (SUV_max_ of 8.05 vs. 3.44, *P*:0.001 and Sensitivity of 100% vs. 53.85%; *P*: 0.015) in comparison to the corresponding 18F-FDG derived values | |
| [Zhao](https://pubmed.ncbi.nlm.nih.gov/33621587/) /2021 (40) | | [^68^Ga] Ga- DOTA-FAPI-04 (1.8-2.2 MBq/kg)  [^18^F] FDG (3.7-5.55 MBq/kg) | 60/3D (WB) | 21 | Retrospective Sub-cohort Study/Comparison with FDG to evaluate diagnostic performance, potential application for radiotherapy planning as compare to CE-CT and FDG | | Locally-Advanced esophageal malignancy: SCC (20), Adenocarcinoma (1) | SUV_max_, TBR values, and GTV | In comparison with FDG, FAPI revealed significant higher uptake (median SUV_max_) toward primary lesion (16.71 vs. 11.23; *P*: 0.002) and regional LNs (4.75 vs. 7.94; *P*: 0.032)  FAPI PET-based GTV delineation may be served as complementary tool beside CE-CT-based radiotherapy planning  FDG and FAPI up to x40% lesion-length detection was similar to endoscopic findings (P > 0.05)  FAPIx20% threshold is favorable for tumor delineation and could change radiotherapy planning (4/21) | |
| [Zhao](https://pubmed.ncbi.nlm.nih.gov/33792760/)/2021 (41) | | [^68^Ga] Ga- DOTA-FAPI-04 (1.8-2.2 MBq/kg)  [^18^F] FDG (3.7 MBq/kg) | 40/3D (WB) | 45 | Retrospective Sub-cohort Study/Assessment diagnostic application of FAPI for primary staging and recurrence detection in contrast to FDG PET and MRI | | Nasopharyngeal carcinomas (NPC):  Initial staging (39), Recurrence (6) | SUV_max_, GTV and TLG, Total lesion FAPI | Regarding newly-diagnosed Pts, FAPI as compare to FDG findings depicted higher uptake (SUV_max_) in primary lesion (16.18 vs. 10.11, *P* < 0.001), regional lymph node (11.42 vs. 7.37, *P* < 0.001), as well as bone and visceral metastases (6.94 vs. 3.11, *P* < 0.001)  Considering TNM staging; FAPI upgraded 26% of Pts and subsequently treatment plan changing in 18% of Pts  FAPI true-positive rate of recurrence diagnosis is greater than FDG (3 vs 2 Pts). However, FAPI depicted two false-positive result  FAPI in contrast to MRI, upgraded T staging in 4 Pts and may change radiotherapy plan | |
|  | 1. **Non-oncologic Applications** | | | | | | | | |  |
| [Finke](https://pubmed.ncbi.nlm.nih.gov/33718446/)/2021 (42) | | [^68^Ga] Ga-FAPI (122-336 MBq) ∅ | 60/3D (WB) | 26 | Retrospective Study/early detection of ICI-induced myocarditis with oncologic FAPI PET and correlation with cardiac markers, cardiac-MR and biopsy | | 26 Pts on-going ICIs drug with no CAD:  Asymptomatic Pts (23), presumed ICI-induced myocarditis symptoms (3) | SUV_max_ | Higher myocardial activity in suspected Pts to local myocardial remodeling due to immune checkpoint-associated myocarditis compared to the asymptomatic enrolled cases (Median SUV_max_: 1.79 vs. 1.15)  No correlation was noticed between FAPI uptake and MRI enhancement. | |
| [Heckmann](https://scholar.google.com/scholar?hl=en&as_sdt=0,5&q=Relationship+Between+Cardiac+Fibroblast+Activation+Protein+Activity+by+Positron+Emission+Tomography+and+Cardiovascular+Disease&btnG=)/  2020 (43) | | [^68^Ga] Ga-FAPI (122-336 MBq) | 60/3D (WB) | 229  (185 + 44) | Two consecutive Retrospective cohorts /evaluation cardiac uptake in cancer Pts | | 12 solid cancer entities who underwent oncologic FAPI PET, questioned for cardiovascular and metabolic risk factors | SUV_max,_ SUV_mean_, and TBR values | High diffuse FAPI cardiac uptake significantly correlated with cardiovascular risk factors (odds ratio: 4.3, *p*: 0.0029), Metabolic state and inflammatory processes ð  Focal FAPI activity showed only positive significant correlation with known cardiovascular state (OR: 3.8 *p*: 0.0008) and Aspirin or statins intake (OR,4.8, *p*: 0.0069)  Whereas negative correlation with Female Gender (OR, 0.13, *p*: 0.0059) | |
| [Luo](http://jnm.snmjournals.org/content/early/2020/06/05/jnumed.120.244723.abstract)/  2020 (44) | | [^68^Ga] Ga-FAPI (55.5-162.8 MBq) ∅  [^18^F] FDG (5.55 MBq/kg) | 40-80/3D (WB) | 26 | Prospective cohort /FAPI vs. FDG PET | | Clinically confirmed Ig-G_4_ RD with different organ involvements | SUV_max_, SUV_mean_ | Significant greater FAPI uptake (SUV_max)_ compared to that of FDG PET in involved organs, e.g., pancreas (15.22 Vs 4.19, *p*<0.0001), bile duct/liver (9.42 Vs 4.58, *p*:0.0081), and salivary gland (8.26 Vs 4.88, *p*:0.0008)  Also, higher detection rate in pancreas (100% Vs 57.9%, *p*:0.0078), bile duct/liver (100% Vs 66.7%, *p*:0.480), and lacrimal gland (100% Vs 71.4%, *p*:0.125)  Higher FAPI detection rate in patient-based analysis (100% Vs 92.3%)  No uptake in FDG-positive LNs | |
| [Notohamiprodjo](https://pubmed.ncbi.nlm.nih.gov/33860458/)/2021 (45) | | [^68^Ga] Ga-FAPI-04 (156 MBq) | Up to 60/  Dynamic Cardiac PET/MR | 5 | Retrospective Study/ draw a comparison between a patient with STEMI after 6 days and control non-CAD patients regarding remodeling and fibrosis states by FAPI PET/Cardiac MR | | Control group: history of cancer with no cardiac disease (4), Acute coronary syndrome (one-vessel disease) (1) | SUV_max,_ SUV_mean_, TBR values | Normal myocardium showed as intense as blood pool (background) activity (Mean SUV_max_ 1.2 Vs 1.5)  A Focal zone of intense FAPI uptake (SUV_max_ :10.3, blood pool :1.4) in the affected myocardium (confirmed by cardiac MR and coronary angiography) with some over-estimation of extension of infarction | |
| [Schmidkonz](https://ard.bmj.com/content/early/2020/07/20/annrheumdis-2020-217408)/  2020 (46) | | [^68^Ga] Ga-FAPI-04 (122-336 MBq)  [^18^F] FDG (3.7 MBq/kg) | N/A | 27 | Case series /Discriminating between fibrotic and inflammatory activity in Ig-G_4_ RD Pts before and after treatment | | Histopathologically proven disease | SUV_max_, SUV_mean_ | FAPI PET imaging could differentiate between fibrotic activity and inflammatory process.  No correlation among FDG and FAPI PET results (SUV_max_) was seen (R=0.1114, *p*:0.6044)  Positive correlation (R=0.9811, *p*<0.0001) was observed between changing in FAPI uptake and lesion volume in CT images in follow-up imaging | |
| [Siebermair](https://link.springer.com/article/10.1007/s12350-020-02307-w)/  2020 (47) | | [^68^Ga] Ga-DOTA-FAPI-04 (140 ± 24 MBq) | 12 ± 7/ 3D (WB) | 32 | Retrospective cohort /Cardiac uptake in FAPI PET/CT and PET/MR and its association with cardio-vascular risk factors | | Oncologic Pts undergoing FAPI PET before anticancer treatment. | SUV_max,_ SUV_mean_, TBR values | Six Pts revealed significantly increased focal cardiac uptake compared to remainder cohort (SUV_max_7.1 vs. 1.5, *P*<0.05)  Significant correlation of known history of CAD (50.0% vs. 0.0%, *P*< 0.01), older age (70.8 vs. 56.0 years, *P*: 0.03), and lower LVEF (46.0 vs. 60.1, *P*: 0.03) with focal high cardiac uptake  Participants without localized uptake showed neither history of CAD nor MI (*p* <0.01 and *p*: 0.03, respectively) | |
| [Zhang](https://pubmed.ncbi.nlm.nih.gov/33452634/)/2021 (48) | | [^68^Ga] Ga-FAPI-04 (N/A) [103]  [^18^F] FDG (N/A) [4] | N/A/ PET/MR | 103 | Retrospective Study/ Investigation of non-malignant focal pancreatic uptake in Pts with different indications | | 7 Pts with various pathologies and incidental focal uptake in pancreas corresponding to FDG (4/7) or anatomical modalities | SUV_max_ | Benign pancreatic lesions including pseudo cysts, areas of prior pancreatitis, and sites of IgG4-related disease showed FAPI uptake with SUVmax of 3.1 to 9.1. However, neither FDG activity nor malignant-appearing anatomical changes were noticed in these lesions  Combination MR acquisition with PET may diminish false-positive findings | |
| [Zhou](https://pubmed.ncbi.nlm.nih.gov/33829416/)/2021 (49) | | [^68^Ga] Ga-FAPI-04 (1.85-2.59 MBq/Kg) | 50-60/3D (WB) | 13 | Retrospective Study/Investigation of diagnostic utility of FAPI in renal fibrosis and compare to biopsy as a gold standard | | Chronic renal failure:  Mild Fibrosis (5), Moderate Fibrosis (5) and Severe Fibrosis (3) | SUV_max,_ SUV_mean_, and TBR values | 12 out of 13 Pts revealed elevated tracer activity and SUV_max_ correlated significantly (*p*: 0.039) with degree of renal fibrosis (3.92 ± 1.50 mild, 5.98 ± 1.68 moderate and 7.67 ± 2.23 severe degree) | |

¶ Full-text was not published

∂ negative, minimal uptake or difficult to discriminate from the adjacent tissue. No exact definition is available for inconclusive FDG PET findings.

Ω not mentioned in detail (not peer reviewed)

§ Two patients had double primary lesions each.

* Available only for 6 patients who underwent FAPI-02 scanning. FAPI imaging showed better visualization and lower background activity in the brain, liver, and oral–laryngeal mucosa in contrast to FDG PET imaging (diagnostic accuracy study was not performed).

η Demandable uptake in the brain, lymph node and delineation between tumor and myocardium in both ^18^F- and [^68^Ga] Ga-FAPI-74 PET were observed.

∅ The paper has not clearly mentioned which FAPI ligand has been used in the study.

ð e.g., Obesity (OR, 2.6, P=0.023), Diabetes mellitus (OR, 2.9, P=0.041), Platinum derivatives chemotherapy drugs (OR, 3.0, P=0.034), prior chest radiotherapy (OR, 3.5, P=0.024), hypothyroidism (OR, 8.6, P=0.012)

**Abbreviations**: AIF, Aluminum Flouride; ACC, Adenoid Cystic Carcinoma; ADC, Apparent Diffusion Coefficient; CAD, Coronary Artery Disease; CE-CT, Contrast-Enhanced Computed Tomography; CI, Conventional Imaging; CRC, Colorectal cancer; CUP, Cancer with Unknown Primary, DTC, Differentiated Thyroid Cancer; FAPI, Fibroblast-Activation Protein Inhibitor; FDG, Flourodeoxyglucose; MBq, Mega Becquerel; HL, Hodgkin lymphoma; NHL, Non- Hodgkin lymphoma; GI, Gastrointestinal; GTV, Gross Tumor Volume; H&N: Head and Neck; HCC, Hepatocellular Carcinoma; ICC, Intrahepatic Cholangiocarcinoma; IDH, Isocitrate Dehydrogenase; Ig-G_4_ RD, Ig-G_4_ related disease; IHC, Immunohistochemistry; LVEF, Left Ventricular Ejection Fraction; LN, Lymph Node; MTC, Medullary Thyroid Cancer; NPV, Negative Predictive Value; NSCLC, Non-Small Cell Lung Cancer, PTRT, Peptide-Targeted Radionuclide Therapy; PPV, Positive Predictive Value; Pt(s), Patient(s); rCBV, relative Cerebral Blood Volume; RCC, Renal Cell Carcinoma; SCC, Squamous Cell Carcinoma; SUV, Standardized Uptake Value; TBR, Tumor-Background Ratio; NET, Neuroendocrine Tumor; ICI, Checkpoint inhibitor; CAD, coronary artery disease; TLG, Total Lesion Glycolysis.

References:

1. Bal C, Roesch F, Ballal S, Yadav M, Tripathi M, Yadav D, et al. 68Ga-DATA-FAPi-05: Biodistribution and Comparison with 18F-FDG PET/CT in Various Cancers. Soc Nuclear Med; 2020.

2. Ballal S, Yadav MP, Moon ES, Kramer VS, Roesch F, Kumari S, et al. Biodistribution, pharmacokinetics, dosimetry of [(68)Ga]Ga-DOTA.SA.FAPi, and the head-to-head comparison with [(18)F]F-FDG PET/CT in patients with various cancers. European journal of nuclear medicine and molecular imaging. 2020.

3. Baum RP, Schuchardt C, Singh A, Chantadisai M, Robiller FC, Zhang J, et al. Feasibility, Biodistribution and Preliminary Dosimetry in Peptide-Targeted Radionuclide Therapy (PTRT) of Diverse Adenocarcinomas using (177)Lu-FAP-2286: First-in-Human Results. J Nucl Med. 2021.

4. Chen H, Pang Y, Wu J, Zhao L, Hao B, Wu J, et al. Comparison of [(68)Ga]Ga-DOTA-FAPI-04 and [(18)F] FDG PET/CT for the diagnosis of primary and metastatic lesions in patients with various types of cancer. European journal of nuclear medicine and molecular imaging. 2020;47(8):1820-32.

5. Chen H, Zhao L, Ruan D, Pang Y, Hao B, Dai Y, et al. Usefulness of [(68)Ga]Ga-DOTA-FAPI-04 PET/CT in patients presenting with inconclusive [(18)F]FDG PET/CT findings. European journal of nuclear medicine and molecular imaging. 2021;48(1):73-86.

6. Dendl K, Koerber SA, Finck R, Mokoala KMG, Staudinger F, Schillings L, et al. (68)Ga-FAPI-PET/CT in patients with various gynecological malignancies. Eur J Nucl Med Mol Imaging. 2021.

7. Ferdinandus J, Kessler L, Hirmas N, Trajkovic-Arsic M, Hamacher R, Umutlu L, et al. Equivalent tumor detection for early and late FAPI-46 PET acquisition. European journal of nuclear medicine and molecular imaging. 2021.

8. Geist BK, Xing H, Wang J, Shi X, Zhao H, Hacker M, et al. Comparison of different kinetic models for dynamic 68Ga-FAPI-04 PET/CT imaging of hepatocellular carcinoma with various, also dual-blood input function. 2020.

9. Giesel FL, Kratochwil C, Schlittenhardt J, Dendl K, Eiber M, Staudinger F, et al. Head-to-head intra-individual comparison of biodistribution and tumor uptake of (68)Ga-FAPI and (18)F-FDG PET/CT in cancer patients. Eur J Nucl Med Mol Imaging. 2021.

10. Giesel FL, Adeberg S, Syed M, Lindner T, Jiménez-Franco LD, Mavriopoulou E, et al. FAPI-74 PET/CT Using Either (18)F-AlF or Cold-Kit (68)Ga Labeling: Biodistribution, Radiation Dosimetry, and Tumor Delineation in Lung Cancer Patients. J Nucl Med. 2021;62(2):201-7.

11. Giesel FL, Kratochwil C, Lindner T, Marschalek MM, Loktev A, Lehnert W, et al. (68)Ga-FAPI PET/CT: Biodistribution and Preliminary Dosimetry Estimate of 2 DOTA-Containing FAP-Targeting Agents in Patients with Various Cancers. Journal of nuclear medicine : official publication, Society of Nuclear Medicine. 2019;60(3):386-92.

12. Guo W, Pang Y, Yao L, Zhao L, Fan C, Ke J, et al. Imaging fibroblast activation protein in liver cancer: a single-center post hoc retrospective analysis to compare [(68)Ga]Ga-FAPI-04 PET/CT versus MRI and [(18)F]-FDG PET/CT. European journal of nuclear medicine and molecular imaging. 2020.

13. Jin X, Wei M, Wang S, Wang G, Lai Y, Shi Y, et al. Detecting fibroblast activation proteins in lymphoma using (68)Ga-FAPI PET/CT. J Nucl Med. 2021.

14. Kessler L, Ferdinandus J, Hirmas N, Bauer S, Dirksen U, Zarrad F, et al. Ga-68-FAPI as diagnostic tool in sarcoma: Data from the FAPI-PET prospective observational trial. J Nucl Med. 2021.

15. Koerber SA, Finck R, Dendl K, Uhl M, Lindner T, Kratochwil C, et al. Novel FAP ligands enable improved imaging contrast in sarcoma patients due to FAPI-PET/CT. Eur J Nucl Med Mol Imaging. 2021.

16. Koerber SA, Staudinger F, Kratochwil C, Adeberg S, Haefner MF, Ungerechts G, et al. The Role of (68)Ga-FAPI PET/CT for Patients with Malignancies of the Lower Gastrointestinal Tract: First Clinical Experience. Journal of nuclear medicine : official publication, Society of Nuclear Medicine. 2020;61(9):1331-6.

17. Komek H, Can C, Guzel Y, Oruc Z, Gundogan C, Yildirim OA, et al. (68)Ga-FAPI-04 PET/CT, a new step in breast cancer imaging: a comparative pilot study with the (18)F-FDG PET/CT. Ann Nucl Med. 2021;35(6):744-52.

18. Kratochwil C, Flechsig P, Lindner T, Abderrahim L, Altmann A, Mier W, et al. (68)Ga-FAPI PET/CT: Tracer Uptake in 28 Different Kinds of Cancer. Journal of nuclear medicine : official publication, Society of Nuclear Medicine. 2019;60(6):801-5.

19. Kuyumcu S, Kovan B, Sanli Y, Buyukkaya F, Has Simsek D, Özkan ZG, et al. Safety of Fibroblast Activation Protein–Targeted Radionuclide Therapy by a Low-Dose Dosimetric Approach Using 177Lu-FAPI04. Clinical nuclear medicine. 2021;Publish Ahead of Print.

20. Liermann J, Syed M, Ben-Josef E, Schubert K, Schlampp I, Sprengel SD, et al. Impact of FAPI-PET/CT on Target Volume Definition in Radiation Therapy of Locally Recurrent Pancreatic Cancer. Cancers. 2021;13(4).

21. Lindner T, Altmann A, Krämer S, Kleist C, Loktev A, Kratochwil C, et al. Design and Development of (99m)Tc-Labeled FAPI Tracers for SPECT Imaging and (188)Re Therapy. Journal of nuclear medicine : official publication, Society of Nuclear Medicine. 2020;61(10):1507-13.

22. Linz C, Brands RC, Kertels O, Dierks A, Brumberg J, Gerhard-Hartmann E, et al. Targeting fibroblast activation protein in newly diagnosed squamous cell carcinoma of the oral cavity - initial experience and comparison to [(18)F]FDG PET/CT and MRI. Eur J Nucl Med Mol Imaging. 2021.

23. Meyer C, Dahlbom M, Lindner T, Vauclin S, Mona C, Slavik R, et al. Radiation Dosimetry and Biodistribution of (68)Ga-FAPI-46 PET Imaging in Cancer Patients. Journal of nuclear medicine : official publication, Society of Nuclear Medicine. 2020;61(8):1171-7.

24. Pang Y, Zhao L, Luo Z, Hao B, Wu H, Lin Q, et al. Comparison of (68)Ga-FAPI and (18)F-FDG Uptake in Gastric, Duodenal, and Colorectal Cancers. Radiology. 2021;298(2):393-402.

25. Qin C, Shao F, Gai Y, Liu Q, Ruan W, Liu F, et al. (68)Ga-DOTA-FAPI-04 PET/MR in the evaluation of gastric carcinomas: comparison with (18)F-FDG PET/CT. Journal of nuclear medicine : official publication, Society of Nuclear Medicine. 2021.

26. Qin C, Liu F, Huang J, Ruan W, Liu Q, Gai Y, et al. A head-to-head comparison of (68)Ga-DOTA-FAPI-04 and (18)F-FDG PET/MR in patients with nasopharyngeal carcinoma: a prospective study. European journal of nuclear medicine and molecular imaging. 2021.

27. Ristau J, Giesel FL, Haefner MF, Staudinger F, Lindner T, Merkel A, et al. Impact of Primary Staging with Fibroblast Activation Protein Specific Enzyme Inhibitor (FAPI)-PET/CT on Radio-Oncologic Treatment Planning of Patients with Esophageal Cancer. Molecular imaging and biology. 2020;22(6):1495-500.

28. Rohrich M, Syed M, Liew DP, Giesel FL, Liermann J, Choyke PL, et al. (68)Ga-FAPI-PET/CT improves diagnostic staging and radiotherapy planning of adenoid cystic carcinomas - Imaging analysis and histological validation. Radiother Oncol. 2021;160:192-201.

29. Röhrich M, Floca R, Loi L, Adeberg S, Windisch P, Giesel FL, et al. FAP-specific PET signaling shows a moderately positive correlation with relative CBV and no correlation with ADC in 13 IDH wildtype glioblastomas. European journal of radiology. 2020;127:109021.

30. Röhrich M, Loktev A, Wefers AK, Altmann A, Paech D, Adeberg S, et al. IDH-wildtype glioblastomas and grade III/IV IDH-mutant gliomas show elevated tracer uptake in fibroblast activation protein-specific PET/CT. European journal of nuclear medicine and molecular imaging. 2019;46(12):2569-80.

31. Röhrich M, Naumann P, Giesel FL, Choyke P, Staudinger F, Wefers A, et al. Impact of (68)Ga-FAPI-PET/CT imaging on the therapeutic management of primary and recurrent pancreatic ductal adenocarcinomas. Journal of nuclear medicine : official publication, Society of Nuclear Medicine. 2020.

32. Serfling S, Zhi Y, Schirbel A, Lindner T, Meyer T, Gerhard-Hartmann E, et al. Improved cancer detection in Waldeyer's tonsillar ring by (68)Ga-FAPI PET/CT imaging. Eur J Nucl Med Mol Imaging. 2021;48(4):1178-87.

33. Shi X, Xing H, Yang X, Li F, Yao S, Congwei J, et al. Comparison of PET imaging of activated fibroblasts and (18)F-FDG for diagnosis of primary hepatic tumours: a prospective pilot study. Eur J Nucl Med Mol Imaging. 2020.

34. Shi X, Xing H, Yang X, Li F, Yao S, Zhang H, et al. Fibroblast imaging of hepatic carcinoma with (68)Ga-FAPI-04 PET/CT: a pilot study in patients with suspected hepatic nodules. European journal of nuclear medicine and molecular imaging. 2021;48(1):196-203.

35. Syed M, Flechsig P, Liermann J, Windisch P, Staudinger F, Akbaba S, et al. Fibroblast activation protein inhibitor (FAPI) PET for diagnostics and advanced targeted radiotherapy in head and neck cancers. European journal of nuclear medicine and molecular imaging. 2020;47(12):2836-45.

36. Wang S, Zhou X, Xu X, Ding J, Liu S, Hou X, et al. Clinical translational evaluation of Al(18)F-NOTA-FAPI for fibroblast activation protein-targeted tumour imaging. Eur J Nucl Med Mol Imaging. 2021.

37. Wang S, Zhou X, Xu X, Ding J, Liu T, Jiang J, et al. Dynamic PET/CT Imaging of (68)Ga-FAPI-04 in Chinese Subjects. Frontiers in oncology. 2021;11:651005.

38. Windisch P, Röhrich M, Regnery S, Tonndorf-Martini E, Held T, Lang K, et al. Fibroblast Activation Protein (FAP) specific PET for advanced target volume delineation in glioblastoma. Radiotherapy and oncology : journal of the European Society for Therapeutic Radiology and Oncology. 2020;150:159-63.

39. Zhao L, Pang Y, Luo Z, Fu K, Yang T, Zhao L, et al. Role of [68 Ga] Ga-DOTA-FAPI-04 PET/CT in the evaluation of peritoneal carcinomatosis and comparison with [18 F]-FDG PET/CT. European Journal of Nuclear Medicine and Molecular Imaging. 2021:1-12.

40. Zhao L, Chen S, Chen S, Pang Y, Dai Y, Hu S, et al. (68)Ga-fibroblast activation protein inhibitor PET/CT on gross tumour volume delineation for radiotherapy planning of oesophageal cancer. Radiotherapy and oncology : journal of the European Society for Therapeutic Radiology and Oncology. 2021;158:55-61.

41. Zhao L, Pang Y, Zheng H, Han C, Gu J, Sun L, et al. Clinical utility of [(68)Ga]Ga-labeled fibroblast activation protein inhibitor (FAPI) positron emission tomography/computed tomography for primary staging and recurrence detection in nasopharyngeal carcinoma. European journal of nuclear medicine and molecular imaging. 2021.

42. Finke D, Heckmann MB, Herpel E, Katus HA, Haberkorn U, Leuschner F, et al. Early Detection of Checkpoint Inhibitor-Associated Myocarditis Using (68)Ga-FAPI PET/CT. Frontiers in cardiovascular medicine. 2021;8:614997.

43. Heckmann MB, Reinhardt F, Finke D, Katus HA, Haberkorn U, Leuschner F, et al. Relationship Between Cardiac Fibroblast Activation Protein Activity by Positron Emission Tomography and Cardiovascular Disease. Circulation Cardiovascular imaging. 2020;13(9):e010628.

44. Luo Y, Pan Q, Yang H, Peng L, Zhang W, Li F. Fibroblast Activation Protein-Targeted PET/CT with (68)Ga-FAPI for Imaging IgG4-Related Disease: Comparison to (18)F-FDG PET/CT. Journal of nuclear medicine : official publication, Society of Nuclear Medicine. 2021;62(2):266-71.

45. Notohamiprodjo S, Nekolla SG, Robu S, Villagran Asiares A, Kupatt C, Ibrahim T, et al. Imaging of cardiac fibroblast activation in a patient after acute myocardial infarction using (68)Ga-FAPI-04. Journal of nuclear cardiology : official publication of the American Society of Nuclear Cardiology. 2021.

46. Schmidkonz C, Rauber S, Atzinger A, Agarwal R, Götz TI, Soare A, et al. Disentangling inflammatory from fibrotic disease activity by fibroblast activation protein imaging. Annals of the rheumatic diseases. 2020;79(11):1485-91.

47. Siebermair J, Köhler MI, Kupusovic J, Nekolla SG, Kessler L, Ferdinandus J, et al. Cardiac fibroblast activation detected by Ga-68 FAPI PET imaging as a potential novel biomarker of cardiac injury/remodeling. Journal of nuclear cardiology : official publication of the American Society of Nuclear Cardiology. 2020.

48. Zhang X, Song W, Qin C, Liu F, Lan X. Non-malignant findings of focal (68)Ga-FAPI-04 uptake in pancreas. European journal of nuclear medicine and molecular imaging. 2021.

49. Zhou Y, Yang X, Liu H, Luo W, Liu H, Lv T, et al. Value of [(68)Ga]Ga-FAPI-04 imaging in the diagnosis of renal fibrosis. European journal of nuclear medicine and molecular imaging. 2021.
