# Supplementary material for: Could Fibroblast Activation Protein (FAP)-Specific Radioligands Be Considered as Pan-Tumor Agents?
Source: Contrast Media Mol Imaging. 2022 Feb 22;2022:3948873. doi: 10.1155/2022/3948873 (PMC8888077; doi:10.1155/2022/3948873)
Supplement: Supplementary Materials — Supplementary Table 1: summary of clinical studies on FAP-tracers. Supplementary Table 2: characteristics of FAP-tracers. Supplementary Table 3: summary of case reports on FAP-tracers. [file 3948873.f1.zip › 3948873.f1/Supplementary table 2 (1).docx]

Table 2. Characteristics of FAP-tracers.

| FAPI Radio-tracer (biomarker) | Advantages | Disadvantages | Oncologic | Non-oncologic | References |
| --- | --- | --- | --- | --- | --- |
| [^68^Ga] Ga-DOTA-FAPI-02 | High binding specificity to the FAP target, low background activity | Tumor washout rate of 75% from 1 to 3 h P.i | Breast, Head and Neck, CUP, Colorectal, Hepatocellular, Liposarcoma, Pancreatic, Prostate, Renal cell carcinoma, Thyroid, NSCLC, Esophageal, Gynecological, IDH-mutant Glioma, Adenoid cystic carcinoma | Not reported yet | (1-7) |
| [^68^Ga] Ga-DOTA-FAPI-04 | High binding specificity to the FAP target, low background activity | Tumor washout rate of 25% from 1 to 3 h P.i | Different primary and metastatic cancers as well as unknown primary carcinomas ^*^, lymphoma, sarcoma, oral SCC | Focal cardiac uptake in cancer Pts ^§^, Ig-G_4_ RD, low-uptake benign hepatic lesion | (1-4, 6-27) |
| [^68^Ga] Ga-NOTA-FAPI-04 |  |  | A patient suspicious for GI malignancy (Primary Lymphoma), lung cancer, pancratic cancer, colorectal cancer,  prostate cancer and lymphoma patients | Not reported yet | (28) (29) |
| [^68^Ga] Ga-DATA-FAPI-05 | Labeling at room temperatures, |  | Different cancers ^Φ^ | Not reported yet | (30) |
| [^68^Ga] Ga-FAPI-46 | Higher tumor uptake with comparable background activity in comparison to FAPI-04, improved tumor retention time, background rapid washout rate | Tumor washout rate of 14% | GI, H&N, Breast, pancreas, Cholangiocellular carcinoma, sarcoma, gynecological tumors, Adenoid cystic carcinoma | Elastofibroma (in a SCC Pt), Bilateral breast uptake due to hormonal change, Post-partum changes in thyroid, breast and uterus | (5-7, 13, 16, 20, 25, 31-35) |
| [^68^Ga] Ga-NOTA-FAPI-74 |  |  | Lung cancer, H&N, pancreato-biliary, GI, gynecological tumors, Adenoid cystic carcinoma | Not reported yet | (5, 6) |
| [^18^F] AIF-NOTA-FAPI-74 |  | Non-significant lower tumor uptake compared to FAPI-04, moderately higher blood pool activity compared to FAPI-04, definable blood vessels until 3h P.i | Lung cancer (adenocarcinoma, squamous cell carcinoma) | Not reported yet | (5) |
| [^18^F] AlF-NOTA-FAPI-04 |  |  | Metastatic breast cancer | Not reported yet | (36) |
| [^68^Ga] Ga-DOTA-SA-FAPi | Improved tumor retention time, enable to link with therapeutic radionuclides, background rapid washout rate | Tumor washout rate of 5.4 to 44.3% | Breast, Lung, H &N, GI, GU, myeloproliferative, Neuroblastoma, GBM, Neurofibroblastoma, Unknown primary carcinoma | Not reported yet | (37) |
| [^177^Lu] Lu-DOTA-SA-FAPi |  |  | End-stage Breast cancer | Not reported yet | (38) |
| [^177^Lu] Lu-DOTA-FAP-2286 | Improved tumor retention time until 10 days P.i |  | Metastatic adenocarcinoma; pancreatic, breast, ovarian and colorectal | Not reported yet | (39, 40) |
| [^68^Ga] Ga-DOTA-FAP-2286 | Enable to link with therapeutic radionuclides |  | Metastatic adenocarcinoma; pancreatic, breast, ovarian and colorectal | Not reported yet | (39, 40) |
| [^99m^Tc] Tc-FAPI-34 | Diagnostic scintigraphy in where PET imaging is not available, enable to link with therapeutic radionuclides |  | Metastatic Ovarian and Pancreatic cancers | Not reported yet | (41) |
| [^153^Sm] Sm-FAPI-46 |  |  | Metastatic soft-tissue sarcoma refractory to routine treatment | Not reported yet | (42) |
| [^90^ Y] Y-FAPI-46 |  |  | Metastatic soft-tissue sarcoma refractory to routine treatment,  Metastatic Ovarian and Pancreatic cancers | Not reported yet | (41, 42) |

^*^ e.g., metastatic and primary lower GI, lung, hepatocellular, breast, cholangiocarcinoma, head and neck, gastric, pancreatic and ovarian cancers, IDH-mutant & UDH-Wildtype gliomas, Waldeyer’s tonsillar carcinomas, sarcoma, NET, Prostate, Desmoid, Chordoma, MTC, ACC, Pheochromocytoma, and DTC

^§^ revealed correlation of focal uptake with cardiovascular risk factors

^Φ^ Not mentioned in detail

**Abbreviations:** CUP, cancer of unknown primary; IDH, Isocitrate Dehydrogenase; Ig-G_4_ RD, Ig-G_4_ related disease; NSCLC, non-small cell lung cancer; Pts, patients; ACC, Adenoid Cystic Carcinoma; DTC, Differentiated Thyroid Cancer; MTC, Medullary Thyroid Cancer; NET, Neuroendocrine Tumor.

References:

1. Giesel FL, Kratochwil C, Lindner T, Marschalek MM, Loktev A, Lehnert W, et al. (68)Ga-FAPI PET/CT: Biodistribution and Preliminary Dosimetry Estimate of 2 DOTA-Containing FAP-Targeting Agents in Patients with Various Cancers. Journal of nuclear medicine : official publication, Society of Nuclear Medicine. 2019;60(3):386-92.

2. Röhrich M, Loktev A, Wefers AK, Altmann A, Paech D, Adeberg S, et al. IDH-wildtype glioblastomas and grade III/IV IDH-mutant gliomas show elevated tracer uptake in fibroblast activation protein-specific PET/CT. European journal of nuclear medicine and molecular imaging. 2019;46(12):2569-80.

3. Röhrich M, Floca R, Loi L, Adeberg S, Windisch P, Giesel FL, et al. FAP-specific PET signaling shows a moderately positive correlation with relative CBV and no correlation with ADC in 13 IDH wildtype glioblastomas. European journal of radiology. 2020;127:109021.

4. Windisch P, Röhrich M, Regnery S, Tonndorf-Martini E, Held T, Lang K, et al. Fibroblast Activation Protein (FAP) specific PET for advanced target volume delineation in glioblastoma. Radiotherapy and oncology : journal of the European Society for Therapeutic Radiology and Oncology. 2020;150:159-63.

5. Rohrich M, Syed M, Liew DP, Giesel FL, Liermann J, Choyke PL, et al. (68)Ga-FAPI-PET/CT improves diagnostic staging and radiotherapy planning of adenoid cystic carcinomas - Imaging analysis and histological validation. Radiother Oncol. 2021;160:192-201.

6. Giesel FL, Kratochwil C, Schlittenhardt J, Dendl K, Eiber M, Staudinger F, et al. Head-to-head intra-individual comparison of biodistribution and tumor uptake of (68)Ga-FAPI and (18)F-FDG PET/CT in cancer patients. Eur J Nucl Med Mol Imaging. 2021.

7. Dendl K, Koerber SA, Finck R, Mokoala KMG, Staudinger F, Schillings L, et al. (68)Ga-FAPI-PET/CT in patients with various gynecological malignancies. Eur J Nucl Med Mol Imaging. 2021.

8. Siebermair J, Köhler MI, Kupusovic J, Nekolla SG, Kessler L, Ferdinandus J, et al. Cardiac fibroblast activation detected by Ga-68 FAPI PET imaging as a potential novel biomarker of cardiac injury/remodeling. Journal of nuclear cardiology : official publication of the American Society of Nuclear Cardiology. 2020.

9. Kratochwil C, Flechsig P, Lindner T, Abderrahim L, Altmann A, Mier W, et al. (68)Ga-FAPI PET/CT: Tracer Uptake in 28 Different Kinds of Cancer. Journal of nuclear medicine : official publication, Society of Nuclear Medicine. 2019;60(6):801-5.

10. Chen H, Pang Y, Wu J, Zhao L, Hao B, Wu J, et al. Comparison of [(68)Ga]Ga-DOTA-FAPI-04 and [(18)F] FDG PET/CT for the diagnosis of primary and metastatic lesions in patients with various types of cancer. European journal of nuclear medicine and molecular imaging. 2020;47(8):1820-32.

11. Shi X, Xing H, Yang X, Li F, Yao S, Zhang H, et al. Fibroblast imaging of hepatic carcinoma with (68)Ga-FAPI-04 PET/CT: a pilot study in patients with suspected hepatic nodules. European journal of nuclear medicine and molecular imaging. 2021;48(1):196-203.

12. Chen H, Zhao L, Ruan D, Pang Y, Hao B, Dai Y, et al. Usefulness of [(68)Ga]Ga-DOTA-FAPI-04 PET/CT in patients presenting with inconclusive [(18)F]FDG PET/CT findings. European journal of nuclear medicine and molecular imaging. 2021;48(1):73-86.

13. Koerber SA, Staudinger F, Kratochwil C, Adeberg S, Haefner MF, Ungerechts G, et al. The Role of (68)Ga-FAPI PET/CT for Patients with Malignancies of the Lower Gastrointestinal Tract: First Clinical Experience. Journal of nuclear medicine : official publication, Society of Nuclear Medicine. 2020;61(9):1331-6.

14. Luo Y, Pan Q, Yang H, Peng L, Zhang W, Li F. Fibroblast Activation Protein-Targeted PET/CT with (68)Ga-FAPI for Imaging IgG4-Related Disease: Comparison to (18)F-FDG PET/CT. Journal of nuclear medicine : official publication, Society of Nuclear Medicine. 2021;62(2):266-71.

15. Schmidkonz C, Rauber S, Atzinger A, Agarwal R, Götz TI, Soare A, et al. Disentangling inflammatory from fibrotic disease activity by fibroblast activation protein imaging. Annals of the rheumatic diseases. 2020;79(11):1485-91.

16. Röhrich M, Naumann P, Giesel FL, Choyke P, Staudinger F, Wefers A, et al. Impact of (68)Ga-FAPI-PET/CT imaging on the therapeutic management of primary and recurrent pancreatic ductal adenocarcinomas. Journal of nuclear medicine : official publication, Society of Nuclear Medicine. 2020.

17. Guo W, Pang Y, Yao L, Zhao L, Fan C, Ke J, et al. Imaging fibroblast activation protein in liver cancer: a single-center post hoc retrospective analysis to compare [(68)Ga]Ga-FAPI-04 PET/CT versus MRI and [(18)F]-FDG PET/CT. European journal of nuclear medicine and molecular imaging. 2020.

18. Shi X, Xing H, Yang X, Li F, Yao S, Congwei J, et al. Comparison of PET imaging of activated fibroblasts and (18)F-FDG for diagnosis of primary hepatic tumours: a prospective pilot study. Eur J Nucl Med Mol Imaging. 2020.

19. Serfling S, Zhi Y, Schirbel A, Lindner T, Meyer T, Gerhard-Hartmann E, et al. Improved cancer detection in Waldeyer's tonsillar ring by (68)Ga-FAPI PET/CT imaging. Eur J Nucl Med Mol Imaging. 2021;48(4):1178-87.

20. Ristau J, Giesel FL, Haefner MF, Staudinger F, Lindner T, Merkel A, et al. Impact of Primary Staging with Fibroblast Activation Protein Specific Enzyme Inhibitor (FAPI)-PET/CT on Radio-Oncologic Treatment Planning of Patients with Esophageal Cancer. Molecular imaging and biology. 2020;22(6):1495-500.

21. Pang Y, Zhao L, Luo Z, Hao B, Wu H, Lin Q, et al. Comparison of (68)Ga-FAPI and (18)F-FDG Uptake in Gastric, Duodenal, and Colorectal Cancers. Radiology. 2021;298(2):393-402.

22. Qin C, Shao F, Gai Y, Liu Q, Ruan W, Liu F, et al. (68)Ga-DOTA-FAPI-04 PET/MR in the evaluation of gastric carcinomas: comparison with (18)F-FDG PET/CT. Journal of nuclear medicine : official publication, Society of Nuclear Medicine. 2021.

23. Wang S, Zhou X, Xu X, Ding J, Liu T, Jiang J, et al. Dynamic PET/CT Imaging of (68)Ga-FAPI-04 in Chinese Subjects. Frontiers in oncology. 2021;11:651005.

24. Jin X, Wei M, Wang S, Wang G, Lai Y, Shi Y, et al. Detecting fibroblast activation proteins in lymphoma using (68)Ga-FAPI PET/CT. J Nucl Med. 2021.

25. Koerber SA, Finck R, Dendl K, Uhl M, Lindner T, Kratochwil C, et al. Novel FAP ligands enable improved imaging contrast in sarcoma patients due to FAPI-PET/CT. Eur J Nucl Med Mol Imaging. 2021.

26. Komek H, Can C, Guzel Y, Oruc Z, Gundogan C, Yildirim OA, et al. (68)Ga-FAPI-04 PET/CT, a new step in breast cancer imaging: a comparative pilot study with the (18)F-FDG PET/CT. Ann Nucl Med. 2021;35(6):744-52.

27. Linz C, Brands RC, Kertels O, Dierks A, Brumberg J, Gerhard-Hartmann E, et al. Targeting fibroblast activation protein in newly diagnosed squamous cell carcinoma of the oral cavity - initial experience and comparison to [(18)F]FDG PET/CT and MRI. Eur J Nucl Med Mol Imaging. 2021.

28. Wang G, Jin X, Zhu H, Wang S, Ding J, Zhang Y, et al. (68)Ga-NOTA-FAPI-04 PET/CT in a patient with primary gastric diffuse large B cell lymphoma: comparisons with [(18)F] FDG PET/CT. European journal of nuclear medicine and molecular imaging. 2021;48(2):647-8.

29. Wang S, Zhou X, Xu X, Ding J, Liu S, Hou X, et al. Clinical translational evaluation of Al(18)F-NOTA-FAPI for fibroblast activation protein-targeted tumour imaging. Eur J Nucl Med Mol Imaging. 2021.

30. Bal C, Roesch F, Ballal S, Yadav M, Tripathi M, Yadav D, et al. 68Ga-DATA-FAPi-05: Biodistribution and Comparison with 18F-FDG PET/CT in Various Cancers. Soc Nuclear Med; 2020.

31. Meyer C, Dahlbom M, Lindner T, Vauclin S, Mona C, Slavik R, et al. Radiation Dosimetry and Biodistribution of (68)Ga-FAPI-46 PET Imaging in Cancer Patients. Journal of nuclear medicine : official publication, Society of Nuclear Medicine. 2020;61(8):1171-7.

32. Hayrapetian A, Girgis MD, Yanagawa J, French SW, Schelbert HR, Auerbach MS, et al. Incidental Detection of Elastofibroma Dorsi With 68Ga-FAPI-46 and 18F-FDG PET/CT in a Patient With Esophageal Cancer. Clinical nuclear medicine. 2021;46(2):e86-e7.

33. Sonni I, Lee-Felker S, Memarzadeh S, Quinn MM, Mona CE, Lückerath K, et al. 68 Ga-FAPi-46 diffuse bilateral breast uptake in a patient with cervical cancer after hormonal stimulation. European journal of nuclear medicine and molecular imaging. 2020:1-3.

34. Dendl K, Koerber SA, Adeberg S, Röhrich M, Kratochwil C, Haberkorn U, et al. Physiological FAP-activation in a postpartum woman observed in oncological FAPI-PET/CT. European journal of nuclear medicine and molecular imaging. 2021.

35. Kessler L, Ferdinandus J, Hirmas N, Bauer S, Dirksen U, Zarrad F, et al. Ga-68-FAPI as diagnostic tool in sarcoma: Data from the FAPI-PET prospective observational trial. J Nucl Med. 2021.

36. Jiang X, Wang X, Shen T, Yao Y, Chen M, Li Z, et al. FAPI-04 PET/CT Using [(18)F]AlF Labeling Strategy: Automatic Synthesis, Quality Control, and In Vivo Assessment in Patient. Frontiers in oncology. 2021;11:649148.

37. Ballal S, Yadav MP, Moon ES, Kramer VS, Roesch F, Kumari S, et al. Biodistribution, pharmacokinetics, dosimetry of [(68)Ga]Ga-DOTA.SA.FAPi, and the head-to-head comparison with [(18)F]F-FDG PET/CT in patients with various cancers. European journal of nuclear medicine and molecular imaging. 2020.

38. Ballal S, Yadav MP, Kramer V, Moon ES, Roesch F, Tripathi M, et al. A theranostic approach of [(68)Ga]Ga-DOTA.SA.FAPi PET/CT-guided [(177)Lu]Lu-DOTA.SA.FAPi radionuclide therapy in an end-stage breast cancer patient: new frontier in targeted radionuclide therapy. European journal of nuclear medicine and molecular imaging. 2021;48(3):942-4.

39. Baum R, Chantadisai M, Smerling C, Schuchardt C, SINGH A, Eismant A, et al. <strong>Peptide-Targeted Radionuclide Therapy (PTRT) using Lu-177 FAP-2286 in Diverse Adenocarcinomas: Feasibility, Biodistribution and Preliminary Dosimetry in a First-in-human study</strong>. Journal of Nuclear Medicine. 2020;61(supplement 1):633-.

40. Baum RP, Schuchardt C, Singh A, Chantadisai M, Robiller FC, Zhang J, et al. Feasibility, Biodistribution and Preliminary Dosimetry in Peptide-Targeted Radionuclide Therapy (PTRT) of Diverse Adenocarcinomas using (177)Lu-FAP-2286: First-in-Human Results. J Nucl Med. 2021.

41. Lindner T, Altmann A, Krämer S, Kleist C, Loktev A, Kratochwil C, et al. Design and Development of (99m)Tc-Labeled FAPI Tracers for SPECT Imaging and (188)Re Therapy. Journal of nuclear medicine : official publication, Society of Nuclear Medicine. 2020;61(10):1507-13.

42. Kratochwil C, Giesel FL, Rathke H, Fink R, Dendl K, Debus J, et al. [153 Sm] Samarium-labeled FAPI-46 radioligand therapy in a patient with lung metastases of a sarcoma. European journal of nuclear medicine and molecular imaging. 2021:1-3.
