# Supplementary material for: Could Fibroblast Activation Protein (FAP)-Specific Radioligands Be Considered as Pan-Tumor Agents?
Source: Contrast Media Mol Imaging. 2022 Feb 22;2022:3948873. doi: 10.1155/2022/3948873 (PMC8888077; doi:10.1155/2022/3948873)
Supplement: Supplementary Materials — Supplementary Table 1: summary of clinical studies on FAP-tracers. Supplementary Table 2: characteristics of FAP-tracers. Supplementary Table 3: summary of case reports on FAP-tracers. [file 3948873.f1.zip › 3948873.f1/Supplementary table 3 (1).docx]

Table 3. Interesting case reports on radiolabeled FAPI PET/CT imaging.

| **First author/**  **Year (Reference)** | **Radiotracer** | **Case Presentation** | **Main finding(s)/Pitfall(s)** |
| --- | --- | --- | --- |
| 1. **Malignant/Oncologic Findings** | | | |
| [**Hao**](https://scholar.google.com/scholar?hl=en&as_sdt=0%2C5&q=68+Ga-FAPI+PET%2FCT+in+Assessment+ofLeptomeningealMetastasesin+a+Patient+With+LungAdenocarcinoma&btnG=)**/2020 (1)** | [^68^Ga] Ga-FAPI  [^18^F] FDG | A lung adenocarcinoma suspected for brain metastasis. Contrast-enhanced MRI revealed leptomeningeal metastasis while FDG PET was negative. FAPI PET conducted for further evaluation. | Abnormal FAPI uptake in the region of brain corresponded to MRI findings.  FDG PETwas negative regarding brain metastasis.  Follow-up FAPI scan showed response to treatment in primary and metastatic lesions. |
| [**Fan**](https://pubmed.ncbi.nlm.nih.gov/32804764/)**/2021 (2)** | [^68^Ga] Ga-FAPI  [^18^F] FDG | Evaluation FDG vs FAPI PET in a gastric signet-ring cell carcinoma. | FAPI demonstrated more metastatic involvement and better visualization of primary lesion. |
| [**Jiang**](https://pubmed.ncbi.nlm.nih.gov/33577199/)**/2020 (3)** | [^68^Ga] Ga-FAPI  [^18^F] FDG | A male subject with slow-growth cutaneous lesion regarding suspicion for malignancy FDG PET was done. Owing to FDG-avid lesions, investigational FAPI PET was performed. | Multiple FDG-avid lesions in perineum, pelvic LNs and facial bones showed higher uptake in FAPI PET.  Biopsy revealed extramammary Paget known as a rare skin intraepithelial adenocarcinoma.  Facial bone uptake proved to an inflammatory process. |
| [**Kratochwil**](https://link.springer.com/article/10.1007/s00259-021-05273-8)**/2021 (4)** | [^153^Sm] Sm-FAPI-46  [^90^ Y] Y-FAPI-46 | A metastatic soft-tissue sarcoma refractory to established-standard treatments underwent radionuclide trial therapy with three cycles of Samarium and radiolabeled Yttrium. | Proper tumor retention and good clearance from normal tissues reaching stable disease for 3 months.  Insufficient specific activity and possible contamination of radiolabeled [^154^Eu] Eu seems to be the main limitations. |
| [**Khreish**](https://pubmed.ncbi.nlm.nih.gov/31814067/)**/2019 (5)** | [^68^Ga] Ga-FAPI-04  [^18^F] FDG  [^68^Ga] Ga-PSMA-11 | A mCRPC patient referred for RLT with ^177^Lu-PSMA and due to disease progression during therapy, restaging with Ga-PSMA PET and FDG PET was done and indicated additional lesion detection in FDG scan (FDG positive/PSMA negative pattern).  Ga-FAPI performed and revealed intense uptake in all-detected lesions. | No FDG positive/FAPI negative or PSMA positive/FAPI negative lesions were noticed.  Although no FAPI positive/FDG negative uptake was present (could be due to dedifferentiated prostate cancer), potential implication of RLT with FAPI could be considerable. |
| [**Giesel**](https://scholar.google.com/scholar?hl=en&as_sdt=0%2C5&q=FAPI-PET%2FCT+improves+staging+in+a+lung+cancer+patient+with+cerebral+metastasis&btnG=)**/2019 (6)** | [^68^Ga] Ga-FAPI-04  [^18^F] FDG | A patient with lung adenocarcinoma underwent FDG PET for staging and revealed lung and mediastinal LN involvement.  Ga-FAPI PET was done to comparison with FDG PET. | Due to cerebral metastases detected by Ga-FAPI (in contrast to FDG findings), TNM up-staging of the patient is noticed. |
| [**Wang**](https://link.springer.com/article/10.1007/s00259-020-04946-0)**/2020 (7)** | [^68^Ga] Ga-NOTA-FAPI-04  [^18^F] FDG | A patient suspicious for gastric malignancy upon CT findings (gastric wall thickening and regional LAP). Asked for FDG PET and trial of FAPI scan (as comparison with FDG PET).  Tissue sampling was also performed and revealed gastric lymphoma. | In the gastric wall lesion, mild uptake of FAPI served more than low-grade FDG uptake (SUV _max_; 5 vs 2.9, respectively).  Meanwhile the regional LNs showed less FAPI uptake than FDG (SUV _max_; 1.4 compared to 2, respectively). |
| [**Chen**](https://journals.lww.com/nuclearmed/Abstract/2020/06000/68Ga_FAPI_PET_CT_Improves_Therapeutic_Strategy_by.16.aspx?context=LatestArticles)**/2020 (8)** | [^68^Ga] Ga-FAPI  [^18^F] FDG | Detection of second primary cancer (lung adenocarcinoma) in a confirmed rectal cancer patient by Ga-FAPI, while FDG PET revealed pleural nodules and lung nodule with low uptake. | FAPI uptake of the lung and pleural nodules was much higher than FDG in this patient (SUV _max_; 7.4 vs 1.5, respectively).  FAPI PET could be more effective than FDG in detection of pulmonary lesions. |
| [**Komek**](file:///C:\Users\hessam\Desktop\FAPI%20Pubmed&%20Scopus\Manuscript%20\55)**/2020 (9)** | [^68^Ga] Ga-FAPI  [^68^Ga] Ga-DOTATATE | A biopsy-proven G2-NET patient underwent Ga-DOTATATE imaging for assessment. The scan revealed primary pancreatic site with multiple liver and lymph node metastases. Ga-FAPI was administered for comparison. | Compared to Ga-DOTATATE, FAPI depicted better liver involvement with good TBR as well as acceptable primary site and lymph node visualization. |
| [**Wu**](https://pubmed.ncbi.nlm.nih.gov/33674912/)**/2021 (10)** | [^68^Ga] Ga-FAPI  [^18^F] FDG | Assessment of PTC metastatic lesions in an Iodine-negative patient with elevated thyroglobulin | Beside pulmonary and mediastinal metastases which were detected by FDG, FAPI revealed higher uptake with visualization of hepatic, bone and abdominal nodal metastases. |
| [**Fu**](https://link.springer.com/article/10.1007/s00259-020-05155-5#article-info)**/2021 (11)** | [^68^Ga] Ga-FAPI-04  [^18^F] FDG | A young patient with gatrointestinal-symptoms committed colonoscopy and confirmed with signet-ring cell carcinoma of the sigmoid colon. In order to staging and further evaluation FDG PET and FAPI PET were acquired. | FDG PET showed hypermetabolism in the primary site, as well as retroperitoneal LNs and peritoneal involvement with low uptake.  FAPI detected more lesions with much intense uptake than FDG. |
| [**Zhao**](https://pubmed.ncbi.nlm.nih.gov/33782310/)**/2021 (12)** | [^68^Ga] Ga-FAPI  [^18^F] FDG | Primary staging with FDG and FAPI PET was done in a pancreatic cancer patient (acinar type). Moreover, abnormal findings in omentum were noticed.  Cytoreductive surgery confirmed the diagnosis. | Primary tumor revealed uptake in both tracers.  Peritoneal involvement seen better with FAPI.  Follow-up FAPI scan after surgery confirmed treatment response. |
| [**Zhao**](https://scholar.google.com/scholar?hl=en&as_sdt=0%2C5&q=Cardiac+angiosarcoma+detected+using+68+Ga-fibroblast+activation+protein+inhibitor+positron+emission+tomography%2Fmagnetic+resonance&btnG=)**/2020 (13)** | [^68^Ga] Ga-FAPI  [^18^F] FDG | FDG PET was done in a patient with unexplained bloody pericardial effusion (suspicious for malignancy). Since in-conclusive findings, Ga-FAPI PET was observed. | FAPI PET revealed abnormal focus of uptake in the right hemi-thorax corresponding to right atrium (SUV_max_ :5.5 & TBR: 3.9) with no other abnormal uptake. However, no obvious uptake was observed in FDG PET.  Cardiac MRI and subsequent biopsy confirmed cardiac angiosarcoma. |
| [**Zhao**](https://scholar.google.com/scholar?hl=en&as_sdt=0%2C5&q=%5B68+Ga%5DGa-DOTA-FAPI-04+improves+tumor+staging+and+monitors+early+response+to+chemoradiotherapy+in+a+patient+with+esophageal+cancer&btnG=)**/2020 (14)** | [^68^Ga] Ga-DOTA-FAPI-04  [^18^F] FDG | Esophageal SCC for primary staging and treatment planning. | Stage upgrading: LN detection by Ga-FAPI PET over FDG PET.  Pulmonary lesion most likely due to post-radiation fibrosis observed by Ga-FAPI PET. |
| [**Deng**](https://pubmed.ncbi.nlm.nih.gov/33630809/)**/2021 (15)** | [^68^Ga] Ga-FAPI  [^18^F] FDG | Lesion detection with FDG and FAPI PET in a doubtful pancreatic tumor. | FDG found mild metabolic activity in primary lesion and bone while no uptake in liver lesions.  FAPI confirmed findings above with much more uptake and revealed liver metastases with increased uptake. |
| [**Liu**](https://link.springer.com/article/10.1007/s00259-020-04997-3)**/2020 (16)** | [^68^Ga] Ga-FAPI-04  [^18^F] FDG | A known case of esophageal SCC during initial MRI staging showed a mass in the pancreas. Further FDG PET and Ga-FAPI for precise evaluation were done and represented metastatic involvement of pancreas (proven by biopsy) and LNs. | Compared to FDG, FAPI PET showed higher tumor-to-background contrast in primary lesion and pancreatic metastasis (SUV _max_; 12.8 and 20.8 vs 6.4 and 12.9, respectively).  Additional detection rate and more uptake of LN involvement.  Small LNs with no FAPI uptake but mild uptake in FDG PET, considered as inflammatory process. |
| [**Ballal**](https://link.springer.com/article/10.1007/s00259-020-04990-w)**/2020 (17)** | [68Ga] Ga-DOTA.SA.FAPI  [177Lu] Lu-DOTA.SA.FAPI | Theranostic concept in a refractory-to-treatment case of breast cancer (IDC) with IHC report of Her2neu positive, ER and PR negative, and FDG findings corresponded to multiple metastatic lesions (loco-regional, liver, lung, bones and newly diagnosed brain lesion) | Ga-FAPI PET detected matched lesions with FDG PET scan.  One cycle of Lu-FAPI RLT revealed proper uptake in all lesions concordantly to Ga-FAPI PET with no adverse effect and within normal limits laboratory data during 4-week follow-up period. |
| [**Wu**](https://pubmed.ncbi.nlm.nih.gov/33417343/)**/2021(18)** | [^68^Ga] Ga-DOTA-FAPI-04 | A middle-aged patient with edema and lymphadenopathy suspected for malignancy.  FAPI PET was performed in order to oncologic purposes. Biopsy confirmed FAPI findings, | Multiple zones of FAPI uptake in skeleton, LNs and soft tissue. Biopsy from FAPI-avid lesions revealed surprisingly, Erdheim-Chester Disease (rare kind of non-Langerhans cells histiocytosis). |
| [**Fu**](https://pubmed.ncbi.nlm.nih.gov/33156049/)**/2020 (19)** | [^68^Ga] Ga-FAPI  [^18^F] FDG | FDG PET was done in the known case of lung cancer with left hip pain. Despite multiple FDG-avid lesion throughout the body, a doubtful brain lesion was observed. Ga-FAPI PET was performed for comparison to inconclusive finding of FDG PET. | FAPI imaging showed better visualization of brain metastasis in comparison with FDG findings |
| [**Guo**](https://scholar.google.com/scholar?hl=en&as_sdt=0%2C5&q=8Ga+FAPI+PET%2FCT+Imaging+in+Peritoneal+Carcinomatosis&btnG=)**/2020 (20)** | [^68^Ga] Ga-FAPI  [^18^F] FDG | Elevated CEA level with normal endoscopy with no signs of malignancy. FDG PET was acquired to help for detection of probable gastrointestinal malignancy.  Also, Ga-FAPI PET was done for comparison. | FDG PET showed multiple mesenteric foci of moderate uptake and failed to detect the primary site.  Ga-FAPI revealed abnormal uptake corresponding to gastric wall with much more abnormal uptake in abdomen.  Tissue sampling confirm gastric adenocarcinoma with peritoneal carcinomatosis. |
| [**Jiang**](https://pubmed.ncbi.nlm.nih.gov/33816303/)**/2021** **(21)** | [^18^F]AlF-NOTA-FAPI-04  [^18^F] FDG | In vivo evaluation of recently developed [^18^F] F-labeled FAPI targeting in a metastatic breast cancer patient and compare to FDG PET (part of an original preclinical study). | The known liver metastasis was found with AlF-FAPI with better visualization in contrast to FDG. |
| [**Yang**](https://pubmed.ncbi.nlm.nih.gov/33860881/)**/2021 (22)** | [^68^Ga] Ga-FAPI | A known case of hypothyroidism with Hashimoto-background presented due to new-onset dyspnea and thyroid enlargement.  In order to possible malignant transformation of thyroid, FAPI PET was performed.  Surgery confirmed the diagnosis. | An enlarged retrosternal bulky thyroid gland with diffuse FAPI uptake was confined to primary thyroid lymphoma. |
| [**Pang**](https://journals.lww.com/nuclearmed/Abstract/2020/08000/68Ga_FAPI_PET_CT_Detects_Gastric_Signet_Ring_Cell.11.aspx)**/2020 (23)** | [^68^Ga] Ga-FAPI  [^68^Ga] Ga-  PSMA  [^99m^Tc] Tc-MDP | Known case of prostate cancer referred for recurrence, revealed super scan. Ga-FAPI PET performed for detecting second primary. | Beside skeletal uptake, Ga-FAPI PET observed gastric wall intense uptake. Histopathology diagnosed gastric cancer.  Bilateral adrenal uptake in Ga-FAPI PET explained by previous hormone therapy-induced inflammation |
| [**Pang**](https://journals.lww.com/nuclearmed/Abstract/2020/11000/68Ga_FAPI_Outperforms_18F_FDG_PET_CT_in.25.aspx)**/2020 (24)** | [^68^Ga] Ga-FAPI  [^18^F] FDG | A known case of infiltrating lobular breast cancer discovered multiple bone metastases and peritoneal carcinomatosis during surveillance with FDG PET. Ga-FAPI PET study performed for comparison. | FAPI demonstrated greater number of bone metastases and much more uptake in peritoneal and omental metastases in comparison with FDG PET. |
| [**Pang**](https://journals.lww.com/nuclearmed/Abstract/2020/07000/Comparison_of_68Ga_FAPI_and_18F_FDG_PET_CT_in_a.21.aspx)**/2020 (25)** | [^68^Ga] Ga-FAPI  [^18^F] FDG | Comparing FDG PET with Ga-FAPI PET for primary staging in a patient with hepatic mass (cholangiocellular carcinoma) and multiple bone and LN metastases. | Compared to FDG PET, Ga-FAPI revealed better visualization and delineation of primary tumor (SUV _max_ 23.2 vs 7.8), more detection rate of metastatic lesions (especially bone metastases) |
| [**Pang**](https://pubmed.ncbi.nlm.nih.gov/33315682/)**/2020 (26)** | [^68^Ga] Ga-FAPI  [^18^F] FDG | A patient with osseous lesions (probably metastases) was evaluated for metastasis work-up. FDG PET was done and demonstrated bone lesions with uptake and failure to discover primary site. Ga-FAPI was acquired for further evaluation. | In comparison with FDG PET, FAPI showed better contrast and higher uptake in the corresponded bony lesion.  Surprisingly FAPI presented intense uptake in the inferior pole of the kidney which was proved to chromophobe RCC |
| [**Zhang**](https://pubmed.ncbi.nlm.nih.gov/33512949/)**/2021 (27)** | [^68^Ga] Ga-FAPI  [^18^F] FDG | A patient with neurologic manifestations underwent MRI, FDG PET and investigational FAPI PET. | A suspicious CNS mass in MRI with obvious edema (suggestive for malignancy) and increased uptake of FDG and FAPI (mild-moderate uptake).  Pathology revealed Primary CNS diffuse large B-cell lymphoma. |
| 1. **Benign/Non-oncologic Findings** | | | |
| [**Hayrapetian**](https://pubmed.ncbi.nlm.nih.gov/32701818/)**/2021 (28)** | [^68^Ga] Ga-FAPI-46  [^18^F] FDG | Performing FDG and FAPI PET in order to initial staging of esophageal SCC. | Both tracers revealed increased uptake in primary tumor with no distant metastasis.  Incidentally FAPI PET showed a sub-scapular mass with high uptake and mild FDG uptake. Biopsy confirmed elastofibroma dorsi |
| [**Zhang**](https://pubmed.ncbi.nlm.nih.gov/33826564/)**/2021 (29)** | [^68^Ga] Ga-DOTA-FAPI-04  [^18^F] FDG | A middle-aged man with pain in the thoracic region and a mass in underlying region. FDG PET performed for evaluation of potential malignancy.  FAPI PET was done for precise diagnosis. | In contrast to FDG mild hypermetabolism, FAPI depicted intense avidity in the pleural-based lesion  Tissue sampling was done and reported solitary fibrous tumor. |
| [**Gu**](https://www.ncbi.nlm.nih.gov/pmc/articles/PMC7531500/)**/2020 (30)** | [^68^Ga] Ga-FAPI  [^18^F] FDG | A patient with supraclavicular LAP and non-specific symptoms performed FDG PET for evaluation of possible malignancy and showed multiple uptakes in spleen and LNs.  FAPI PET was performed for complementary assessment.  Histopathological evaluation of supraclavicular lymph node confirmed extrapulmonary tuberculosis. | FAPI images revealed intense uptake in the mentioned lesions with no added observation of other lesions.  Comparing FAPI PET with FDG findings no added value in detection of lesions and differentiation benign form malignant processes were notable.  Considering higher uptake in observed lesions, FAPI could be beneficial in assessment of response to treatment of tuberculosis. |
| [**Hao**](https://scholar.google.com/scholar?hl=en&as_sdt=0%2C5&q=%5B18+F%5DFDG+and+%5B68+Ga%5DGa-DOTA-FAPI-04+PET%2FCT+in+the+evaluation+of+tuberculous+lesions&btnG=)**/2020 (31)** | [^68^Ga] Ga-DOTA-FAPI-04  [^18^F] FDG | A patient with history of pulmonary tuberculosis and recently diagnosed tubercular meningitis (based on MRI observation) underwent FDG PET for assessment of other possible lesions. Also, FAPI PET was done for evaluation inflammation-induced fibrosis activity. | As compared to FDG findings (lung and hilar lesions as well as lumbar spine uptake), FAPI PET demonstrated higher uptake in the mentioned lesions.  Additional three cerebral lesions with high FAPI uptake were detected. |
| [**Can**](https://pubmed.ncbi.nlm.nih.gov/33826569/)**/2021 (32)** | [^68^Ga] Ga-FAPI-04  [^18^F] FDG | FDG and FAPI PET were performed for assessment of a patient with history of breast cancer. | All FAPI uptakes in the primary tumor and LNs was in concordance with FDG findings, except the non-uniform thyroid uptake which was only seen in FAPI PET. Further evaluation and follow-up revealed evidence of thyroiditis. |
| [**Gündoğan**](https://pubmed.ncbi.nlm.nih.gov/33782295/)**/2021 (33)** | [^68^Ga] Ga-FAPI-04  [^18^F] FDG | A known case of breast cancer who underwent FDG and FAPI PET due to incidental finding of FAPI (not FDG) in the same breast. | Primary tumor showed more FAPI uptake than FDG.  Another incidental FAPI uptake in the same breast was benign lymphoid tissue. |
| [**Qin**](https://pubmed.ncbi.nlm.nih.gov/33086278/)**/2020 (34)** | [^68^Ga] Ga-FAPI  [^18^F] FDG | A case of recurrent retroperitoneal angiomyolipoma with low FDG uptake compared to Ga-FAPI scan. | Considering high FAPI uptake in this lesion, Ga-FAPI may be useful in benign tumor imaging. |
| [**Liu**](https://pubmed.ncbi.nlm.nih.gov/33782317/)**/2021 (35)** | [^68^Ga] Ga-FAPI | FAPI PET was done in a colon cancer patient. Incidental uptake in gallbladder and T-spine | No evidence of metastases,  incidental findings: chronic cholecystitis and osteophyte in affected vertebra. |
| [**Liu**](https://pubmed.ncbi.nlm.nih.gov/33826568/)**/2021 (36)** | [^68^Ga] Ga-DOTA-FAPI-04  [^18^F] FDG | Owing to further evaluation of a suspicious pulmonary nodules, FDG and FAPI PET were performed. | Both tracers depicted increased uptake in pulmonary lesions with consolidation (pneumonia).  Unexpectedly FAPI showed intense activity in a splenic lesion (hemangioma) without FDG uptake. |
| [**Xing**](https://link.springer.com/article/10.1007/s12350-020-02517-2)**/2021 (37)** | [^68^Ga] Ga-FAPI  Cardiac-MR | A known case of CTEPh underwent FAPI PET as compared to cardiac MR | High and non-uniform FAPI uptake in the right heart (free wall). FAPI PET may be sensitive imaging than MRI in localizing fibrosis as a prognostic tool. |
| [**Sonni**](https://link.springer.com/article/10.1007/s00259-020-04947-z)**/2020 (38)** | [^68^Ga] Ga-FAPI-46  [^18^F] FDG | A case of invasive cervical carcinoma who received hormonal stimulation therapy underwent FDG PET for initial evaluation. Also, FAPI PET as a trial study was done. | Beside abnormal uptake in primary tumor and pelvic LN involvement as seen in both FDG and FAPI images, the FAPI PET demonstrated other results: bilateral diffuse symmetrical breast uptake which was considered as hormone therapy-induced increase in activity of fibroglandular tissue (not seen on FDG PETI and more intense uptake in uterus (as compared to FDG images) considered as physiologic uptake. |
| [**Wu**](https://pubmed.ncbi.nlm.nih.gov/33661208/)**/2021 (39)** | [^68^Ga] Ga-FAPI  [^18^F] FDG | FDG and FAPI PET for further assessment of incidentally revealed a pulmonary nodule were done. | Pulmonary malignant nodule showed increased uptake with both tracer (biopsy confirmed malignancy).  Beside FDG-avidity in lumbar fracture, surprisingly FAPI revealed intense uptake (a potential pitfall regarding mimicking malignancy). |
| [**Zheng**](https://link.springer.com/article/10.1007/s00259-020-05185-z)**/2021(40)** | [^68^Ga] Ga-FAPI  [^18^F] FDG | A young lady with sign and symptoms of inflammatory myopathy, underwent both FDG PET and FAPI PET scans for assessment of possible synchronous malignancy. Biopsy was done and juvenile polymyositis was confirmed | Multiple uptakes were noted throughout affected muscles (as seen in MRI) in both FDG and FAPI PET. No other abnormal radio-tracer activity was observed all over the rest of the body. |
| [**Dendl**](https://pubmed.ncbi.nlm.nih.gov/33543324/)**/2021 (41)** | [^68^Ga] Ga-FAPI-46 | A known case of head and neck cancer after delivery and during lactation was recruited for oncologic FAPI PET scan. | FAPI PET depicted high uptake in breast bilaterally, uterus and thyroid bed. Sensitive organs to post-partum changes may observed FAP over-expression. |
| [**Wang**](file:///C:\Users\hessam\Desktop\FAPI%20Pubmed&%20Scopus\Manuscript%20\53)**/2020 (42)** | [^68^Ga] Ga-FAPI-04  [^18^F] FDG | An advanced case of head and neck carcinoma underwent both FDG and FAPI PET imaging for staging. | Incidental findings: bilateral homogenous breast FAPI uptake might be due to hormonal changes, |
| [**Wang**](https://link.springer.com/article/10.1007/s12350-020-02407-7#article-info)**/2020 (43)** | [^68^Ga] Ga-FAPI | A confirmed-case with diagnosis of idiopathic pulmonary arterial hypertension. FAPI PET was done as a trial imaging. | FAPI uptake in the right heart which can be served as a prognostic factor |
| [**Zhao**](https://journals.lww.com/nuclearmed/Abstract/2020/10000/68Ga_FAPI_PET_CT_in_Assessment_of_Liver_Nodules_in.30.aspx)**/2020 (44)** | [^68^Ga] Ga-FAPI  [^18^F] FDG | Ga-FAPI PET assessment of multiple liver nodules in a cirrhotic patient with suspicion of hepatocellular carcinoma by contrast-enhanced MRI although FDG PET was negative. | Increased liver parenchymal uptake and relatively lower FAPI uptake in the nodules might be useful for differentiate nodules in cirrhotic liver. The pathology of liver biopsy revealed hepatic adenoma, |
| [**Totzeck**](https://scholar.google.com/scholar?hl=en&as_sdt=0%2C5&q=10.1093%2Feurheartj%2Fehz736&btnG=)**/2020 (45)** | [^68^Ga] Ga-FAPI | A patient with pancreatic cancer (ductal adenocarcinoma) and under systemic anti-cancer treatment was enrolled in Ga-FAPI imaging for oncologic purposes. In addition to primary and metastatic lesion (peritoneal carcinomatosis and liver metastases) detection, an intense uptake in the region of the myocardium was noticed.  The patient declared history of coronary artery disease with no recent symptoms. | FAPI uptake in the myocardium considered as a result of cardiotoxicity due to anti-cancer treatment.  FAPI PET might be reasonable for early detection of myocardial injury after anti-cancer treatment. |
| [**Pan**](file:///C:\Users\hessam\Desktop\FAPI%20Pubmed&%20Scopus\Manuscript%20\54)**/2020 (46)** | [^68^Ga] Ga-FAPI  [^18^F] FDG | Recent hydronephrosis with suspicious retroperitoneal involvement. Marked FDG hypermetabolic activity corresponded to retroperitoneal mass. Ga-FAPI imaging was done for assessment of fibrotic activity. | Ga-FAPI also showed intense uptake with no other abnormality. The patient significantly responded to anti-fibrosis therapy. |
| [**Pan**](https://scholar.google.com/scholar?hl=en&as_sdt=2005&sciodt=0%2C5&cites=14267388535503069840&scipsc=&q=Recurrent+Immunoglobulin+G4%E2%80%93Related+Disease+Shown+on+18+F-FDG+and+68+Ga-FAPI+PET%2FCT&btnG=)**/2020 (47)** | [^68^Ga] Ga-FAPI  [^18^F] FDG | A known case of IgG4 related disease presented with disease recurrence. FDG PET was done and showed multiple abnormal uptakes in the liver, pancreas, prostate, pituitary, submandibular gland, pleural and pericardial regions as well as LNs.  Ga-FAPI was trialed for evaluation of fibrotic activity and disease extension. | In comparison with FDG findings, FAPI detected the same lesions as the FDG-positive, except no uptake in LNs. Additional finding was lacrimal gland tracer accumulation. |
| [**Lin**](https://pubmed.ncbi.nlm.nih.gov/33826575/)**/2021 (48)** | [^68^Ga] Ga-FAPI-04  [^18^F] FDG | A recently diagnosed adenocarcinoma of cardias presented for initial staging and treatment planning. Both FDG and FAPI PET were acquired. | Primary tumor and inflammatory bilateral hilar and mediastinal LNs showed increased avidity in both scans. Nonetheless, in FAPI PET an uptake in the T-spine was observed due to Schmorl node. |
| [**Xu**](https://link.springer.com/article/10.1007/s00259-020-05028-x)**/2020 (49)** | [^68^Ga] Ga-DOTA-FAPI-04 | A patient with history of chronic prostatitis and shoulder degenerative disease presented with new-onset headache and suspicious brain lesion for possible metastasis as seen on MRI. FDG PET and FAPI PET were performed for further evaluation. Subsequent tissue sampling form prostate revealed prostate cancer. | FDG PET imaging showed abnormal uptake in the prostate bed and the affected shoulder (considered as arthritis) with no abnormal brain finding.  FAPI observed the same abnormal activity as the FDG findings with much more uptake in the affected shoulder in addition to brain lesion suggesting a possible application for FAPI PET in inflammation evaluation (arthritis). |
| [**Zhu**](https://pubmed.ncbi.nlm.nih.gov/33086274/)**/2020 (50)** | [^68^Ga] Ga-FAPI-04 | A patient with NET underwent FAPI PET for oncologic purposes. Incidentally increased uptake was observed in the inferior wall of left ventricle cavity. | Considering patient’s history and ECG findings FAPI uptake assumed as indication of old myocardial infarction.  FAPI may be a possible tool for evaluation of cardiac remodeling. |
| [**Lou**](https://link.springer.com/article/10.1007/s00259-020-05129-7)**/2020 (51)** | [^68^Ga] Ga-FAPI  [^18^F] FDG | A case of known Inflammatory bowel disease from years ago (Crohn’s Dis.) and morbid complications. was observed to take both FAPI and FDG scans for evaluation disease status and complications and compared to a case of ulcerative colitis in order to FAPI activity | In both patients FDG revealed increased metabolism. Whereas FAPI only depicted increased activity in the Crohn’s case. |
| [**Y Luo**](https://journals.lww.com/nuclearmed/Abstract/2020/04000/Intense_FAPI_Uptake_in_Inflammation_May_Mask_the.12.aspx)**/2020 (52)** | [^68^Ga] Ga-FAPI  [^18^F] FDG | A patient with enlarged pancreas and nodular lesion in the uncinate process at CT underwent FDG PET for further evaluation. Ga-FAPI was trialed for potential role in differentiation of pancreatic lesions.  Histopathology study showed ductal carcinoma of pancreas with cancer-induced pancreatitis. | Compared to FDG findings, FAPI showed more intense uptake in the pancreas with no delineation of uncinate lesion (inflammation mask underlying lesion). |
| [**Luo**](https://scholar.google.com/scholar?hl=en&as_sdt=0%2C5&q=gG4-related+disease+revealed+by+68+Ga-FAPI+and+18+F-FDG+PET%2FCT&btnG=)**/2019 (53)** | [^68^Ga] Ga-FAPI  [^18^F] FDG | A histopathology-proven case of IgG4 related disease underwent FDG PET study for evaluation with multiple involvement in the parotid, submandibular glands, LNs and lung lesions. FAPI PET was performed in comparison with FDG PET in pre-treatment and post-treatment setting. | Compared to FDG PET findings, FAPI imaging revealed more intense uptake in the parotid, submandibular glands and lung lesions  No FAPI uptake in FDG-positive LNs may be due to low fibrosis.  Post-treatment follow-up FAPI PET demonstrated markedly improvement of the affected lesions. |
| [**Song**](https://pubmed.ncbi.nlm.nih.gov/33883497/)**/2021 (54)** | [^68^Ga] Ga-FAPI | A patient with signet-ring cell carcinoma of stomach was found with a discordant uptake in primary and metastatic lesions at FAPI PET/MR | An uptake in the temporal bone after follow-up and performing other modalities was considered as fibrous dysplasia mimicking bone metastasis. |
| [**Zhou**](https://pubmed.ncbi.nlm.nih.gov/33449295/)**/2021 (55)** | [^68^Ga] Ga-FAPI  [^18^F] FDG | A known case of colon malignancy underwent surgery six months ago and recent FDG PET revealed no abnormality. FAPI PET was performed to further assessment. | FAPI incidentally found high uptake in thyroid bed which was diagnosed as thyroiditis. |

Abbreviations: SCC, Squamous Cell Carcinoma; LAP, Lymphadenopathy; mCRPC, metastatic Castration-Resistant Prostate Cancer; RLT, Radio-Ligand Therapy; IDC, Invasive Ductal carcinoma; CT, Computed Tomography; SUV, Standardized Uptake Value; CTEPh, chronic thromboembolic pulmonary hypertension.

References:

1. Hao B, Wu J, Pang Y, Sun L, Chen H. 68Ga-FAPI PET/CT in Assessment of Leptomeningeal Metastases in a Patient With Lung Adenocarcinoma. Clinical nuclear medicine. 2020;45(10):784-6.

2. Fan C, Guo W, Su G, Chen B, Chen H. Widespread Metastatic Gastric Signet-Ring Cell Carcinoma Shown by 68Ga-FAPI PET/CT. Clinical nuclear medicine. 2021;46(2):e78-e9.

3. Jiang C, Song S. 68Ga-FAPI and 18F-FDG PET/CT in Perineum Extramammary Paget Disease. Clinical nuclear medicine. 2021;46(4):342-4.

4. Kratochwil C, Giesel FL, Rathke H, Fink R, Dendl K, Debus J, et al. [153 Sm] Samarium-labeled FAPI-46 radioligand therapy in a patient with lung metastases of a sarcoma. European journal of nuclear medicine and molecular imaging. 2021:1-3.

5. Khreish F, Rosar F, Kratochwil C, Giesel FL, Haberkorn U, Ezziddin S. Positive FAPI-PET/CT in a metastatic castration-resistant prostate cancer patient with PSMA-negative/FDG-positive disease. European journal of nuclear medicine and molecular imaging. 2020;47(8):2040-1.

6. Giesel FL, Heussel CP, Lindner T, Röhrich M, Rathke H, Kauczor HU, et al. FAPI-PET/CT improves staging in a lung cancer patient with cerebral metastasis. European journal of nuclear medicine and molecular imaging. 2019;46(8):1754-5.

7. Wang G, Jin X, Zhu H, Wang S, Ding J, Zhang Y, et al. (68)Ga-NOTA-FAPI-04 PET/CT in a patient with primary gastric diffuse large B cell lymphoma: comparisons with [(18)F] FDG PET/CT. European journal of nuclear medicine and molecular imaging. 2021;48(2):647-8.

8. Chen H, Zhao L, Ruan D, Sun L, Lin Q. 68Ga-FAPI PET/CT Improves Therapeutic Strategy by Detecting a Second Primary Malignancy in a Patient With Rectal Cancer. Clinical nuclear medicine. 2020;45(6):468-70.

9. Kömek H, Gündoğan C, Can C. 68Ga-FAPI PET/CT Versus 68Ga-DOTATATE PET/CT in the Evaluation of a Patient With Neuroendocrine Tumor. Clinical nuclear medicine. 2021;46(5):e290-e2.

10. Wu J, Ou L, Zhang C. Comparison of (68)Ga-FAPI and (18)F-FDG PET/CT in metastases of papillary thyroid carcinoma. Endocrine. 2021.

11. Fu L, Hu K, Tang G, Wu H, Zhou W. [(68)Ga]Ga-FAPI-04 PET/CT imaging in signet-ring cell carcinoma of sigmoid colon. European journal of nuclear medicine and molecular imaging. 2021.

12. Zhao L, Pang Y, Wei J, Hao B, Chen H. Use of 68Ga-FAPI PET/CT for Evaluation of Peritoneal Carcinomatosis Before and After Cytoreductive Surgery. Clinical nuclear medicine. 2021.

13. Zhao L, Pang Y, Lin Q, Chen H. Cardiac angiosarcoma detected using 68Ga-fibroblast activation protein inhibitor positron emission tomography/magnetic resonance. European heart journal. 2021;42(13):1276.

14. Zhao L, Chen S, Lin L, Sun L, Wu H, Lin Q, et al. [(68)Ga]Ga-DOTA-FAPI-04 improves tumor staging and monitors early response to chemoradiotherapy in a patient with esophageal cancer. European journal of nuclear medicine and molecular imaging. 2020;47(13):3188-9.

15. Deng M, Chen Y, Cai L. Comparison of 68Ga-FAPI and 18F-FDG PET/CT in the Imaging of Pancreatic Cancer With Liver Metastases. Clinical nuclear medicine. 2021.

16. Liu Q, Shi S, Xu X, Yu X, Song S. The superiority of [(68)Ga]-FAPI-04 over [(18)F]-FDG PET/CT in imaging metastatic esophageal squamous cell carcinoma. European journal of nuclear medicine and molecular imaging. 2021;48(4):1248-9.

17. Ballal S, Yadav MP, Kramer V, Moon ES, Roesch F, Tripathi M, et al. A theranostic approach of [(68)Ga]Ga-DOTA.SA.FAPi PET/CT-guided [(177)Lu]Lu-DOTA.SA.FAPi radionuclide therapy in an end-stage breast cancer patient: new frontier in targeted radionuclide therapy. European journal of nuclear medicine and molecular imaging. 2021;48(3):942-4.

18. Wu S, Pang Y, Chen Y, Sun H, Chen H. 68Ga-DOTA-FAPI-04 PET/CT in Erdheim-Chester Disease. Clinical nuclear medicine. 2021;46(3):258-60.

19. Fu W, Liu L, Liu H, Zhou Z, Chen Y. Increased FAPI Uptake in Brain Metastasis From Lung Cancer on 68Ga-FAPI PET/CT. Clinical nuclear medicine. 2021;46(1):e1-e2.

20. Guo W, Chen H. (68)Ga FAPI PET/CT Imaging in Peritoneal Carcinomatosis. Radiology. 2020;297(3):521.

21. Jiang X, Wang X, Shen T, Yao Y, Chen M, Li Z, et al. FAPI-04 PET/CT Using [(18)F]AlF Labeling Strategy: Automatic Synthesis, Quality Control, and In Vivo Assessment in Patient. Frontiers in oncology. 2021;11:649148.

22. Yang X, Gong W, Chen Y. (68)Ga-FAPI PET/CT imaging in a patient with primary thyroid lymphoma. Endocrine. 2021.

23. Pang Y, Huang H, Fu L, Zhao L, Chen H. 68Ga-FAPI PET/CT Detects Gastric Signet-Ring Cell Carcinoma in a Patient Previously Treated for Prostate Cancer. Clinical nuclear medicine. 2020;45(8):632-5.

24. Pang Y, Zhao L, Chen H. 68Ga-FAPI Outperforms 18F-FDG PET/CT in Identifying Bone Metastasis and Peritoneal Carcinomatosis in a Patient With Metastatic Breast Cancer. Clinical nuclear medicine. 2020;45(11):913-5.

25. Pang Y, Hao B, Shang Q, Sun L, Chen H. Comparison of 68Ga-FAPI and 18F-FDG PET/CT in a Patient With Cholangiocellular Carcinoma: A Case Report. Clinical nuclear medicine. 2020;45(7):566-7.

26. Pang Y, Wei J, Shang Q, Zhao L, Chen H. 68Ga-Fibroblast Activation Protein Inhibitor, a Promising Radiopharmaceutical in PET/CT to Detect the Primary and Metastatic Lesions of Chromophobe Renal Cell Carcinoma. Clinical nuclear medicine. 2021;46(2):177-9.

27. Zhang Y, Cai J, Lin Z, Yao S, Miao W. Primary Central Nervous System Lymphoma Revealed by 68Ga-FAPI and 18F-FDG PET/CT. Clinical nuclear medicine. 2021.

28. Hayrapetian A, Girgis MD, Yanagawa J, French SW, Schelbert HR, Auerbach MS, et al. Incidental Detection of Elastofibroma Dorsi With 68Ga-FAPI-46 and 18F-FDG PET/CT in a Patient With Esophageal Cancer. Clinical nuclear medicine. 2021;46(2):e86-e7.

29. Zhang A, Zhang H, Zhou X, Li Z, Li N. Solitary Fibrous Tumors of the Pleura Shown on 18F-FDG and 68Ga-DOTA-FAPI-04 PET/CT. Clinical nuclear medicine. 2021.

30. Gu B, Luo Z, He X, Wang J, Song S. 68Ga-FAPI and 18F-FDG PET/CT Images in a Patient With Extrapulmonary Tuberculosis Mimicking Malignant Tumor. Clinical nuclear medicine. 2020;45(11):865-7.

31. Hao B, Wu X, Pang Y, Sun L, Wu H, Huang W, et al. [(18)F]FDG and ([68)Ga]Ga-DOTA-FAPI-04 PET/CT in the evaluation of tuberculous lesions. European journal of nuclear medicine and molecular imaging. 2021;48(2):651-2.

32. Can C, Gündoğan C, Güzel Y, Kaplan İ, Kömek H. 68Ga-FAPI Uptake of Thyroiditis in a Patient With Breast Cancer. Clinical nuclear medicine. 2021.

33. Gündoğan C, Güzel Y, Can C, Alabalik U, Kömek H. False-Positive 68Ga-Fibroblast Activation Protein-Specific Inhibitor Uptake of Benign Lymphoid Tissue in a Patient With Breast Cancer. Clinical nuclear medicine. 2021.

34. Qin C, Gai Y, Liu Q, Shao F, Lan X. Elevated 68Ga-FAPI Accumulation in a Recurrent Angiomyolipoma. Clinical nuclear medicine. 2020;45(12):1034-5.

35. Liu H, Chen Z, Yang X, Fu W, Chen Y. Increased 68Ga-FAPI Uptake in Chronic Cholecystitis and Degenerative Osteophyte. Clinical nuclear medicine. 2021.

36. Liu H, Wang Y, Zhang W, Cai L, Chen Y. Elevated 68Ga-FAPI Activity in Splenic Hemangioma and Pneumonia. Clinical nuclear medicine. 2021.

37. Xing H-Q, Gong J-N, Chen B-X, Guo X-J, Yang Y-H, Huo L, et al. Comparison of 68 Ga-FAPI imaging and cardiac magnetic resonance in detection of myocardial fibrosis in a patient with chronic thromboembolic pulmonary hypertension. Journal of Nuclear Cardiology. 2021:1-3.

38. Sonni I, Lee-Felker S, Memarzadeh S, Quinn MM, Mona CE, Lückerath K, et al. 68 Ga-FAPi-46 diffuse bilateral breast uptake in a patient with cervical cancer after hormonal stimulation. European journal of nuclear medicine and molecular imaging. 2020:1-3.

39. Wu J, Liu H, Ou L, Jiang G, Zhang C. FAPI Uptake in a Vertebral Body Fracture in a Patient With Lung Cancer: A FAPI Imaging Pitfall. Clinical nuclear medicine. 2021.

40. Zheng J, Chen H, Lin K, Yao S, Miao W. [(68)Ga]Ga-FAPI and [(18)F]FDG PET/CT images in a patient with juvenile polymyositis. European journal of nuclear medicine and molecular imaging. 2021.

41. Dendl K, Koerber SA, Adeberg S, Röhrich M, Kratochwil C, Haberkorn U, et al. Physiological FAP-activation in a postpartum woman observed in oncological FAPI-PET/CT. European journal of nuclear medicine and molecular imaging. 2021.

42. Wang LJ, Zhang Y, Wu HB. Intense Diffuse Uptake of 68Ga-FAPI-04 in the Breasts Found by PET/CT in a Patient With Advanced Nasopharyngeal Carcinoma. Clinical nuclear medicine. 2021;46(5):e293-e5.

43. Wang L, Zhang Z, Zhao Z, Yan C, Fang W. 68 Ga-FAPI right heart uptake in a patient with idiopathic pulmonary arterial hypertension. Journal of Nuclear Cardiology. 2020:1-3.

44. Zhao L, Gu J, Fu K, Lin Q, Chen H. 68Ga-FAPI PET/CT in Assessment of Liver Nodules in a Cirrhotic Patient. Clinical nuclear medicine. 2020;45(10):e430-e2.

45. Totzeck M, Siebermair J, Rassaf T, Rischpler C. Cardiac fibroblast activation detected by positron emission tomography/computed tomography as a possible sign of cardiotoxicity. European heart journal. 2020;41(9):1060-.

46. Pan Q, Luo Y, Zhang W. Idiopathic Retroperitoneal Fibrosis With Intense Uptake of 68Ga-Fibroblast Activation Protein Inhibitor and 18F-FDG. Clinical nuclear medicine. 2021;46(2):175-6.

47. Pan Q, Luo Y, Zhang W. Recurrent Immunoglobulin G4-Related Disease Shown on 18F-FDG and 68Ga-FAPI PET/CT. Clinical nuclear medicine. 2020;45(4):312-3.

48. Lin R, Lin Z, Zhang J, Yao S, Miao W. Increased 68Ga-FAPI-04 Uptake in Schmorl Node in a Patient With Gastric Cancer. Clinical nuclear medicine. 2021.

49. Xu T, Zhao Y, Ding H, Cai L, Zhou Z, Song Z, et al. [(68)Ga]Ga-DOTA-FAPI-04 PET/CT imaging in a case of prostate cancer with shoulder arthritis. European journal of nuclear medicine and molecular imaging. 2021;48(4):1254-5.

50. Zhu W, Guo F, Wang Y, Ding H, Huo L. 68Ga-FAPI-04 Accumulation in Myocardial Infarction in a Patient With Neuroendocrine Carcinoma. Clinical nuclear medicine. 2020;45(12):1020-2.

51. Luo Y, Pan Q, Xu H, Zhang R, Li J, Li F. Active uptake of 68 Ga-FAPI in Crohn’s disease but not in ulcerative colitis. European journal of nuclear medicine and molecular imaging. 2020:1-2.

52. Luo Y, Pan Q, Zhang W, Li F. Intense FAPI Uptake in Inflammation May Mask the Tumor Activity of Pancreatic Cancer in 68Ga-FAPI PET/CT. Clinical nuclear medicine. 2020;45(4):310-1.

53. Luo Y, Pan Q, Zhang W. IgG4-related disease revealed by (68)Ga-FAPI and (18)F-FDG PET/CT. European journal of nuclear medicine and molecular imaging. 2019;46(12):2625-6.

54. Song Y, Qin C, Liu F, Lan X. Fibrous Dysplasia Mimicking Skeletal Metastasis on 68Ga-FAPI PET Imaging. Clinical nuclear medicine. 2021.

55. Zhou Y, He J, Chen Y. (68)Ga-FAPI PET/CT imaging in a patient with thyroiditis. Endocrine. 2021.
